# Plasma Environment, Radiation, Structure, and Evolution of the Uranian System (PERSEUS): A Dedicated Orbiter Mission Concept to Study Space Physics at Uranus

**DOI:** 10.1007/s11214-023-01013-6

**Published:** 2023-10-19

**Authors:** Ian J. Cohen, Evan J. Smith, George B. Clark, Drew L. Turner, Donald H. Ellison, Ben Clare, Leonardo H. Regoli, Peter Kollmann, Daniel T. Gallagher, G. Allan Holtzman, Justin J. Likar, Takeshi Morizono, Matthew Shannon, Kimberly S. Vodusek

**Affiliations:** The Johns Hopkins University Applied Physics Laboratory, Laurel, MD USA

**Keywords:** Uranus, planetary magnetospheres, Ice Giants, space physics, space exploration, mission design

## Abstract

The Plasma Environment, Radiation, Structure, and Evolution of the Uranian System (PERSEUS) mission concept defines the feasibility and potential scope of a dedicated, standalone Heliophysics orbiter mission to study multiple space physics science objectives at Uranus. Uranus’s complex and dynamic magnetosphere presents a unique laboratory to study magnetospheric physics as well as its coupling to the solar wind and the planet’s atmosphere, satellites, and rings. From the planet’s tilted and offset, rapidly-rotating non-dipolar magnetic field to its seasonally-extreme interactions with the solar wind to its unexpectedly intense electron radiation belts, Uranus hosts a range of outstanding and compelling mysteries relevant to the space physics community. While the exploration of planets other than Earth has largely fallen within the purview of NASA’s Planetary Science Division, many targets, like Uranus, also hold immense scientific value and interest to NASA’s Heliophysics Division. Exploring and understanding Uranus’s magnetosphere is critical to make fundamental gains in magnetospheric physics and the understanding of potential exoplanetary systems and to test the validity of our knowledge of magnetospheric dynamics, moon-magnetosphere interactions, magnetosphere-ionosphere coupling, and solar wind-planetary coupling. The PERSEUS mission concept study, currently at Concept Maturity Level (CML) 4, comprises a feasible payload that provides closure to a range of space physics science objectives in a reliable and mature spacecraft and mission design architecture. The mission is able to close using only a single Mod-1 Next-Generation Radioisotope Thermoelectric Generator (NG-RTG) by leveraging a concept of operations that relies of a significant hibernation mode for a large portion of its 22-day orbit.

## Introduction

Uranus, the seventh planet in the solar system and the first planet to be “discovered” by telescopic observation, is an oddity in multiple senses. From its unique magnetospheric configuration to its quiescent atmosphere and heating imbalance to its dense and narrow rings and incredibly dark and tectonized icy satellites, the Uranian system hosts a range of outstanding and compelling mysteries of interest to disciplines across space science. The only in-situ observations of the Uranian magnetosphere to-date are from the 1986 Voyager 2 flyby (e.g., Stone and Miner 1986), which have left us with several outstanding mysteries regarding its magnetospheric dynamics (e.g., Kollmann et al. 2020; Paty et al. 2020). As such, the study of the Uranian magnetosphere is a crucial step for testing the limits of our understanding of magnetospheric dynamics; this is especially important as the community uses its collective knowledge of planets to guide the understanding of even more exotic regimes like exoplanets and brown dwarf stars (e.g., Schrijver 2009; Nichols et al. 2012; Kislyakova et al. 2014; Hallinan et al. 2015).

Uranus, and its sister Ice Giant, Neptune, remain the only two planets in the solar system that have yet to be thoroughly explored by an orbiting mission. These worlds differ from both the rocky terrestrial planets as well as the Gas Giants. Whereas Jupiter and Saturn are made mostly of hydrogen, the bulk compositions of Uranus and Neptune have significant populations of heavier “ices” like water, methane, and ammonia (e.g., Helled and Fortney 2020). The ever-growing list of exoplanets reveals that these Ice Giants may be indicative of the most common size of planets in the galaxy (e.g., Fulton et al. 2017; Bean et al. 2021), but remain the least-investigated planets in the solar system.

One of the Key Science Goals of the 2013 Solar and Space Physics Decadal Survey was to “*Discover and characterize fundamental processes that occur both within the heliosphere and throughout the universe*”. Uranus therefore presents a compelling scientific target for the space physics community because it can “*serve as* [$a$] *cosmic laborator*[$y$] *for studying universal plasma phenomena*” the same way as “*the Sun, the heliosphere, and Earth’s magnetosphere and ionosphere*” (NRC 2013).

The 2013 Decadal Survey emphasized that $``$[$b$]*ecause of the importance of understanding the range of processes operating in the universe, as well as their operation under different environmental conditions, continued progress in comparative magnetospheres is a key objective for the coming decade*” (NRC 2013). Underscoring the compelling nature of the Uranian magnetosphere, the Decadal Survey’s Panel on Solar Wind-Magnetosphere Interactions (SWMI) urged “*NASA’s Heliophysics Division* [$to$] *partner with the Planetary Division to ensure that appropriate magnetospheric instrumentation be fielded on missions to other planets. In particular, the SWMI panel’s highest priority in planetary magnetospheres is a mission to orbit Uranus*” (NRC 2013). The Plasma Environment, Radiation, Structure, and Evolution of the Uranian System (PERSEUS) mission concept presented here was intentionally designed to explicitly investigate the scope, feasibility, and resource demands of a dedicated Heliophysics orbiter mission to explore the magnetosphere of Uranus and its coupling to the solar wind as well as the planet’s atmosphere, satellites, and rings. While borrowing heavily from the recently well-explored trade space of sending an orbiting mission to Uranus (e.g., Arridge et al. 2014; Hubbard 2010; Hofstadter et al. 2017; Leonard et al. 2021; Cohen et al. 2022; Simon et al. 2022), PERSEUS sets itself apart by being the only mission concept specifically dedicated and designed to measure the particles and fields environment and brings a new concept of operations (CONOPS) that maximizes the power efficiency of the flight system. The PERSEUS concept presented here focuses on a baseline point design, which puts it at a CML of 4 (e.g., Wheatcraft and Lewis 2018).

Despite this and the high priority placed on exploration of the Uranian system by both the 2013 Solar and Space Physics and 2022 Planetary Science and Astrobiology Decadal Surveys (NRC 2013; NASEM 2022), it is not assured that the instruments most relevant and appropriate for Heliophysics investigations will be included on a future Planetary Science mission. This is despite the cross-disciplinary benefits of instruments primarily used for planetary magnetospheric investigations (Cohen and Rymer 2020; Kollmann et al. 2020). In particular, although the Voyager, Galileo, Cassini, and Juno missions carried robust particles and fields instrumentation, recent trends in the development of NASA Planetary Science missions have been worrisome as multiple missions over the past few years have included limited such instrumentation as the science objectives more narrowly focus on geology and/or habitability. Fortunately, the current Uranus Orbiter and Probe (UOP) concept prioritized in the recent Planetary Science and Astrobiology Decadal Survey (NASEM 2022) allocated 15.1 kg and 13.1 W (maximum expected values (MEVs)) to the “Fields and Particles Package” and magnetometer. This is ∼20% of the total payload mass allocation and 40% of the total power; for comparison, 36% of the payload mass allocation and 26% of the payload power allocation for Cassini were dedicated to particles and fields instruments, though this also included dust and neutral measurements not baselined for UOP. However, it must be noted that several of the heritage particle instruments baselined in the UOP study have insufficient geometric factors for the Uranian system and thus would require tailoring that will likely demand additional mass resources. Furthermore, it must also be noted that several of the heritage particle instruments also relied on shared electronics that likely result in a systematic underestimate of the required resources for these sensors.

Exploring the Uranian magnetosphere with a properly-instrumented orbiter would provide analogous insight to Uranus as was provided for Earth by the earliest single-spacecraft orbiters with appropriate instrumentation (e.g., the Explorer and ISEE series spacecraft), for Saturn by Cassini, and for Jupiter by Galileo. Perhaps most significantly, a focused Heliophysics orbiter mission to Uranus would complement any future magnetospheric missions to other planets in the solar system, as it would provide an invaluable data point for comparative magnetospheric studies. There is also strong interest from the European community for scientific and possible hardware participation in such a mission (e.g., Arridge et al. 2012; Fletcher et al. 2020, 2022; Blanc et al. 2021) Finally, of course, any such Heliophysics-focused mission would be a natural complement to any future Planetary Science mission to the Uranian system (e.g., Hubbard 2010; Hofstadter et al. 2017; Simon et al. 2022).

## Science Investigation

The magnetospheres of Uranus and Neptune are complex and very different from those found at Jupiter, Saturn, Earth, or Mercury. As Paty et al. (2020) summarize, “*Each of these ice giant planets possess a highly tilted dipolar magnetic moment with a strong quadrupole moment, which, combined with obliquity, create magnetospheres rife with asymmetries and with strong diurnal and seasonal variability. From the constantly shifting regions of reconnection across the magnetopause on daily and seasonal scales, to their helically twisted magnetotails and curved current sheets, the ice giant magnetospheres represent critical departures from Earth’s magnetosphere – upon which the theories of magnetospheric physics have been developed and tested – and implore further study with the questions they raise. Their distant location from the Sun (19.8 and 30.0 AU, respectively) also leads to a significant difference in the local solar wind characteristics at Uranus and Neptune as compared to that experienced at Earth.*”

The Uranian magnetosphere in particular is unique in multiple aspects when compared to the rest of the magnetized planets in the solar system. First and foremost is its extreme obliquity (i.e.,the angle between its rotational axis and the vector normal to the ecliptic plane), which at 98° is by far the most extreme in the solar system and subjects the planet and its magnetosphere to dramatic seasonal variations. As such, Uranus provides an opportunity to explore a uniquely complex and dynamic magnetospheric system with unexpectedly strong electron radiation belts, and extreme diurnally- and seasonally-driven interactions with the solar wind, and a much higher-Mach number bow shock that is unlike shocks at other distances to the Sun.

The 2013 Decadal Survey further detailed that the $``$[k]*ey magnetospheric measurements for a Uranus mission would include magnetic field, plasma waves, plasma, energetic particles, dust and neutral mass spectra, and global images in* [ultraviolet], [infrared], *and* [energetic neutral atoms]” (NRC 2013). It is unlikely that all of these measurements can be made from a smaller-scale Heliophysics-focused mission and thus this study largely focuses on the charged particle and fields measurements outlined above as well as ultraviolet (UV) emissions. While an infrared (IR) imaging instrument would also contribute significantly to compelling space physics science objectives focusing on the planet’s upper atmosphere, this type of sensor is assumed to be high-priority for science objectives targeted by a future Planetary Science mission and is already included in the baseline UOP payload (NRC 2013); therefore it is not included in the baseline payload for PERSEUS. The science questions posed in the Science Traceability Matrix (STM; Table 1) represent a sampling of the outstanding Space physics mysteries at Uranus that PERSEUS can address.

**Table 1:** The Science Traceability Matrix (STM) for PERSEUS, a dedicated space physics mission to orbit Uranus

Science Goal	Science Questions	Physical Parameter	Investigation Objective Requirements		Mission Top Level Requirements
			Measurement (*Italics denote changes from SQ1*)	Requirements	
**Solar and Space Physics Decadal Survey Key Science Goal #4**: Discover and characterize fundamental processes that occur both within the heliosphere and throughout the universe.	[SQ1] How do internal and external sources and drivers shape particle populations and enable particle transport within Uranus’s magnetosphere?	Magnetic field topology	3D vector magnetic field	Range: ±20,000 nT Res: ≤0.1 nT Temporal Res: 10 s	[M1] Orbits covering large ranges of radial distance, MLAT, and local time inside magnetosphere [M2] Electromagnetically clean spacecraft
		Plasma population	Plasma energy, angular, and compositional distributions	Range: ∼10 eV – 30 keV/Q Temporal Res: 10 s Species: e^−^, H^+^, $\mathrm{H}_{2/3}^{+}$, He$^{+/2+}$, C$^{+/5+}$, O$^{+/6+}$	[M1]
		Suprathermal ion sources and dynamics	Suprathermal ion energy, angular, and compositional distributions	Range: ∼1–200 keV/Q Temporal Res: 10 s Species: H^+^, $\mathrm{H}_{2/3}^{+}$, He$^{+/2+}$, C$^{+/5+}$, O$^{+/6+}$	[M1]
		Energetic particle sources and dynamics	Energetic particle energy, angular, and compositional distributions	Range: 30 keV – ≥ 10 MeV Temporal Res: 10 s Species: e^−^, H^+^, He^n+^, C^n+^, O^n+^	[M1]
		Wave activity	Plasma wave power and spectrum	Range: ∼2-10s kHz Temporal Res: ≤5 s	[M1, M2]
	[SQ2] What does Uranus’s global current system look like and how does this couple to upper atmospheric dynamics?	Auroral precipitation	UV auroral emissions	Wavelengths: 121.6 nm (Ly-*α*), 80-160 nm (H_2_) Spatial Res: 1,000 km Temporal Res: 60 s	[M3] Imager pointed towards magnetic polar region [M4] Pointing control: 0.5° [M5] Pointing knowledge: 0.2° [M6] Pointing stability: 0.5°
			Electron energy and angular distributions	Temporal Res: 1 s Angular Res: 15°	[M7] Orbits sampling magnetic polar regions at <10 R_U_
		Auroral currents	3D vector magnetic field	Same as [SQ1]	[M2, M7]
	[SQ3] What particle acceleration processes are present and/or dominant at the Uranian bow shock?	Magnetic field topology	3D vector magnetic field	Range: ±20 nT Res: ≤0.01 nT Temporal Res: 0.05 s	[M2] [M8] Orbits encountering bow shock at varying local times
		Plasma population	Plasma energy, angular, and compositional distributions	Temporal Res: 1 s	[M8]
		Suprathermal ion sources and dynamics	Suprathermal ion energy, angular, and compositional distributions	Temporal Res: 1 s	[M8]
		Energetic particle sources and dynamics	Energetic particle energy, angular, and compositional distributions	Temporal Res: 1 s	[M8]
		Wave activity	Plasma wave power and spectrum	Temporal Res: 0.001 s	[M2, M8]
	[SQ4] Which processes source and shape Uranus’s radiation belts?	Magnetic field topology	Same as [SQ1]	Same as [SQ1]	[M2] [M9] Orbits covering large local time range <10 R_U_
		Plasma population	Same as [SQ1]	Same as [SQ1]	[M9]
		Suprathermal ion sources and dynamics	Same as [SQ1]	Same as [SQ1]	[M9]
		Energetic particle sources and dynamics	Same as [SQ1]	Same as [SQ1]	[M9]
		Wave activity	Same as [SQ1]	Same as [SQ1]	[M2, M9]
	[SQ5] How do interplanetary pickup ionization processes differ at Uranus’s heliocentric distance?	Magnetic field topology	3D vector magnetic field	Range: ±20 nT Res: ≤0.01 nT Temporal Res: 0.01 s	[M2] [M10] Orbits enabling sampling of pristine solar wind upstream of bow shock
		Pickup ion population	Plasma ion energy, angular, and compositional distributions	Range: ∼1 – 10 keV/Q Species: H^+^, He^+^, C^+^, O^+^	[M10]

### Space Physics Science Questions

The PERSEUS mission concept targets five science questions that span the realm of space physics to include topics of relevance to the heliospheric, magnetospheric, and aeronomy communities. Furthermore, these questions significantly expand the space physics focus of this dedicated Heliophysics mission concept compared to the limited magnetospheric questions targeted by the Planetary Science-focused UOP mission (NASEM 2022; Simon et al. 2022).

#### Science Question #1: Magnetospheric Sources, Drivers, and Transport - How do Internal and External Sources and Drivers Shape Particle Populations and Enable Particle Transport Within Uranus’s Magnetosphere?

The magnetosphere of Uranus offers a unique configuration that may hold the missing piece to understand the drivers of magnetospheric dynamics throughout the solar system (e.g., Paty et al. 2020, and references therein). With the planetary spin axis tilted by 98° relative to the ecliptic and a highly-tilted magnetic dipole axis (∼59° with respect to the spin axis), the rapid rotation and continually-changing orientation of the magnetic field presents a dynamic, asymmetrical obstacle to the impinging solar wind (e.g., Cao and Paty 2017, 2021). As such, understanding the general nature of the solar wind-magnetosphere interaction at Uranus is fundamental to gaining insights into the overall drivers and dynamics of the system.

Plasma transport within a planetary magnetosphere may be driven by external and/or internal forces. External forcing would suggest that global plasma convection in Uranus’s magnetosphere is driven primarily by the solar wind (i.e., the Dungey cycle; Dungey 1961), whereas plasma in an internally-driven system would be accelerated and energized by centrifugal forces from the rapidly-rotating planetary magnetic field (i.e., the Vasyliuñas cycle; Vasyliunas 1983). The magnetospheres of terrestrial planets with an intrinsic magnetic field (i.e., Earth and Mercury) are primarily driven by solar wind forcing, whereas the magnetospheres of gas giants Jupiter and Saturn are thought to be dominated by forces driven by internal plasma sources and fast planetary rotation. Voyager 2 observed phenomena, such as substorm-like injection signatures (Mauk et al. 1987) and magnetotail plasmoids (DiBraccio and Gershman 2019), that suggest the presence of magnetic reconnection in the system and that Uranus may be solar wind-driven (e.g., Richardson et al. 1988; Selesnick 1988; Ye and Hill 1994).

Uranus is a fast (∼17-hr) rotator, which alongside the large dipole tilt results in a dynamic and complicated interaction between the solar wind and Uranus’s magnetosphere that varies dramatically over the course of a single Uranian day (e.g., Masters 2014; Cao and Paty 2017) and gives rise to a dynamic helical configuration in the magnetotail (Tóth et al. 2004; Arridge 2015; Griton et al. 2018; Griton and Pantellini 2020). It remains unknown, yet hypothesized, that internal drivers also play some role in global magnetospheric dynamics at Uranus, even though the convection and corotation electric fields are orthogonal near the solstices and may prevent the formation of a traditional plasmapause (Selesnick and Richardson 1986; Vasyliuñas 1986). More recent analyses by Gershman and DiBraccio (2020) and Turner et al. (2023) support the solar wind-driven hypothesis by suggesting that the Uranian system is constantly configured and interacting with favorable solar wind conditions to enable reconnection along the dayside and flank magnetopause, which would likely drive a nearly-continuous global convection system. Although it is believed that the system is solar wind-driven, it remains a mystery why, unlike all other planets, no solar wind alpha particles were found by Voyager 2 at higher energies (Krimigis et al. 1986; Cheng et al. 1987; Kollmann et al. 2020). Masters (2014, 2018) concluded that the conditions at Uranus demand nearly anti-parallel magnetic shear angles for any magnetopause reconnection. Turner et al. (2023) found that there are essentially always locations along the magnetopause with anti-parallel magnetic shear for every spin phase, Uranian season, and interplanetary magnetic field (IMF) orientation. Conversely, Cowley (2013) hypothesized that reconnection along the dayside Uranian magnetosphere might be inhibited by open magnetic field lines that drape over the rapidly rotating system. If Uranus is predominantly rotationally-driven, like Jupiter, then the lack of a clear plasma source (like the influence of Io on Jupiter) could explain the dearth of magnetospheric plasma observed by Voyager 2 (McNutt et al. 1987). However, recent results by Cohen et al. (2023) revealed a source of energetic particles in the inner magnetosphere that is likely originating from lower-energy plasma originating from one or both of the moons Miranda and Ariel.

Investigating the sources, drivers, and transport mechanisms of the Uranian magnetosphere will require multiple orbits covering a broad range of radial distances and L-shells, magnetic local times (MLT), and magnetic latitudes (MLAT). One fortuitous consequence of the tilted dipole and rapid rotation of Uranus is that a spacecraft in an elliptical orbit near the ecliptic plane is able to sample a very large range of radial distances, L-shells, and magnetic latitudes during a single rotation of the planet (see Sect. 3.4). A mission arriving within a decade of the 2050 Uranian equinox would have the chance to observe and compare a different configuration than Voyager 2 as the alignment of the planet’s rotation axis changes seasonally. Necessary measurements include: a broad range of plasma, suprathermal, and energetic particles with composition determination; magnetic fields; and plasma waves. In particular, new measurements must be made but were not obtained by Voyager 2 include coverage of electromagnetic ion cyclotron (EMIC) waves and determination of plasma and suprathermal ion composition (Cohen et al. 2023).

#### Science Question #2: Aurora and Magnetosphere-Ionosphere Coupling - What Does Uranus’s Global Current System Look Like and How Does This Couple to Upper Atmospheric Dynamics?

At Earth, the global current system is known to flow into and out of the ionosphere via the large-scale “Region 1” and “Region 2” field-aligned current (FAC) systems with current closure occurring in the E-region of the ionosphere and the plasma sheet in the magnetotail. These auroral emissions are primarily driven by electron precipitation resulting from the earthward acceleration of particles following from magnetic reconnection that occurs in the magnetotail as part of the solar wind-driven Dungey cycle (Dungey 1961). Conversely, at Jupiter, the main auroral oval is thought to be driven by precipitation resulting from the breakdown of plasma corotation (Clarke 2012) while the origins of the more dynamic emissions poleward of the main oval remains a topic of much debate (e.g., Grodent 2015; Zhang et al. 2021). While it remains unclear how the Uranian magnetosphere-ionosphere current system may be organized and controlled, it is unlikely to be as straightforward as those of Earth given the system’s complex and dynamic magnetic field topology. For example, Voyager 2 observed evidence of a curved current sheet in the magnetotail (Hammond et al. 1990).

Voyager 2 observed auroral emissions at Uranus in the H_2_ Lyman and Lyman-$\alpha $ UV emission bands around both magnetic poles (Broadfoot et al. 1986). These were later followed by Hubble Space Telescope (HST) observations of spotty dayside UV auroral emissions that corotate with the planet (Lamy et al. 2017). These are in stark contrast to the quasi-stable auroral oval features observed at Earth, Jupiter, and Saturn (e.g., Mauk and Bagenal 2012; Grodent 2015). Lamy et al. (2012) attempted to investigate magnetosphere-ionosphere coupling processes by observing the auroral response to expected the passage of a coronal mass ejection (CME). No response was observed, which informed the conclusion that auroral emissions at Uranus are not driven by the solar wind, which could potentially be the result of suppressed dayside reconnection as predicted by Cowley (2013). However, it must be underscored that the analysis relied on significant assumptions on when the CME was anticipated to reach Uranus. New analysis by Turner et al. (2023) suggests that a “monohemispheric” solar wind interaction could result in the spot-like emissions observed by HST while a “reflected interhemispheric” configuration could explain the curved current sheet seen by Voyager 2.

Further interrogating the structure and dynamics of the magnetosphere-ionosphere coupling and auroral processes at play in the Uranian system will require observations of both the auroral currents as well as the particle precipitation. This will require UV imaging of the magnetic poles in the relevant H_2_ Lyman and Ly-$\alpha $ bands as well as in-situ measurements of the precipitating auroral particles at relatively low altitudes along with the magnetic field to both determine pitch angles and estimate FAC intensities.

#### Science Question #3: Shock Acceleration - What Particle Acceleration Processes Are Present and/or Dominant at the Uranian Bow Shock?

Collisionless shocks are a universal physical phenomenon and are known to be a source of particle acceleration (e.g., Balogh and Treumann 2013). Planetary bow shocks form when the supersonic solar wind interacts with both magnetized and unmagnetized planets in the solar system. This provides a wide range of characteristics in these shocks based on the intrinsic characteristics of the planets themselves as well as the evolution of the solar wind throughout the heliosphere. For example, the average solar wind conditions at 1 AU coupled with the nature of Earth’s magnetic field give rise to a bow shock that typically exists across a fairly low range of Mach numbers ($M_{A} < 15$) (e.g., Winterhalter and Kivelson 1998). Given the similar solar wind characteristics experienced by Venus and Mars, their bow shocks are similar to Earth’s, whereas higher-Mach number shocks are typically found at the Giant Planets (e.g., Balogh and Treumann 2013). This means that the bow shocks of the outer planets provide an important stepping stone in enabling the extrapolation of in-situ space physics observations of collisionless shocks to more distant and extreme astrophysical shocks that are inaccessible to in-situ investigation. Multiple processes are known to accelerate particles at collisionless shocks, including - but not limited to - Fermi acceleration (e.g., Fermi 1949; Jokipii 1966), shock surfing (e.g., Sagdeev 1966; Hoshino 2001), diffusive acceleration (e.g., Scholer 1985), wave-particle interactions (e.g., Oka et al. 2006), and shock drift acceleration (e.g., Armstrong 1985). However, it remains unclear which of these processes may be at play at the bow shock of Uranus.

Voyager 2 encountered the Uranian bow shock several times during its 1986 flyby (Bridge et al. 1986; Gurnett et al. 1986; Ness et al. 1986). The first crossing near the sub-solar point was of a very strong (compared to that of Earth; $M_{A} = 23$), quasi-perpendicular shock (Bagenal et al. 1987). Some particular characteristics of the shock were notable. First, the plasma $\beta $ was unusually high (∼3) compared to what was expected for the typical solar wind conditions at 19.8 AU (Bagenal et al. 1987). Second, there was surprisingly low plasma wave activity in the shock foot (Moses et al. 1989). Observations were also obtained of multiple whistler-mode wave types (Smith et al. 1989) as well as upstream energetic ions concluded to have escaped from the magnetosphere (Krimigis et al. 1988). Voyager 2 encounter multiple additional crossings of an oblique, lower-Mach number ($M_{A} = 18$) shock at the flank of the magnetosphere (Smith et al. 1991).

The typical nature of the Uranian bow shock and determination of which acceleration processes occur at it remain unknown. To explore this, an orbital mission will be required that achieves multiple orbits with an apoapsis high enough to regularly encounter the dayside bow shock, which was encountered at 22.5 R_U_ by Voyager 2 (Ness et al. 1986), across as wide a range of local times as possible. Investigation of the acceleration process at play will require plasma, suprathermal, and energetic particle measurements (with mass and charge-state composition, if possible) as well as magnetic field and waves observations. In particular, mass and charge-state determination of the suprathermal and energetic particles will be important to discern between multiple potential acceleration mechanisms (e.g., Turner et al. 2018).

#### Science Question #4: Radiation Belt Dynamics - Which Processes Source and Shape Uranus’s Radiation Belts?

Radiation belts – the regions of magnetospheres that trap and energize particles – provide in-situ laboratories as diverse as the planets they encompass to study the universal processes of particle acceleration. Uranus is especially interesting in this respect because Voyager 2 observations raise questions that significantly challenge our understanding of the processes that ultimately form planetary radiation belts. In order for the particles to accumulate to high intensities, radiation belts need to draw energy rapidly from a large reservoir of lower energy plasma and/or lose the accelerated particles very slowly – i.e., the sources must outweigh any loss processes. Neither appeared to be the case at Uranus, which was observed to have a curiously low plasma density magnetosphere (McNutt et al. 1987) and where observations of intense waves would be expected to lead to both acceleration and efficient particle losses (Millan and Thorne 2007; Tripathi and Singhal 2008).

A particular mystery remains as to why Uranus’s electron belts appear surprisingly intense up to energies of ∼1 MeV (e.g., compared to other Giant planets; Mauk and Fox 2010), while its ion belts show low intensities, despite sharing several physical processes (Mauk 2015). A potential explanation for this may be tied to the intense whistler-mode wave emissions observed in the inner magnetosphere, which were the highest observed by Voyager 2 at any other planet (Kurth and Gurnett 1991). Whistler-mode chorus waves are thought to play a role for both electron acceleration (Reeves et al. 2013; Thorne et al. 2013) and loss (Allison et al. 2019; Shprits et al. 2016), which may have the net effect of explaining the observed high intensities. It is also possible that there are other processes at work, such as extreme drift-resonance pumping of the electrons due to the extreme offset between the rotational and magnetic field axes (e.g., Ukhorskiy et al. 2014; Hao et al. 2021). Unfortunately, Voyager 2 was not able to observe the ion-mode (e.g., EMIC) waves most relevant to both ion radiation belt acceleration (e.g., Wang et al. 2019) and losses (e.g., Usanova et al. 2014; Angelopoulos et al. 2023). However, the possibility of a moon sourcing neutral material into the inner magnetosphere could potentially drive wave growth that may play a significant role in generating and sustaining the radiation belts (Cohen et al. 2023). It remains unclear whether the energetic ion population in the radiation belts sources from pickup ions emanating from a source within the system (e.g., Cheng 1987; Cohen et al. 2023) – as is the case at Jupiter (e.g., Clark et al. 2016, 2020; Szalay et al. 2022) - or from higher charge-state species from the solar wind. However, simulations by Masters et al. (2022) suggest that the dynamics of Uranus’s asymmetric magnetosphere may preferentially drive losses of ≳100 keV protons, which may naturally inhibit the intentsity of its ion radiation belts.

Investigating the sources and dynamics of Uranus’s radiation belts requires multiple orbits covering a broad range of MLT inside of ∼10 R_U_. Measurements of the complete particle population range covering plasma, suprathermal, and energetic particles including mass and charge-state composition will be necessary to determine the potential source(s) seeding the radiation belts. Magnetic field and waves measurements will also be required to track the dynamic motion of the magnetosphere, distinguish trapped particles, as well as investigate potential wave-particle interactions that may accelerate source populations into and/or scatter particles out of the belts.

#### Science Question #5: Interplanetary Pickup Ionization - How do Interplanetary Pickup Ionization Processes Differ at Uranus’s Heliocentric Distance?

Heliospheric pickup ions (PUIs) are generated when incoming interstellar neutrals (primarily originating from the local interstellar cloud) are ionized via plasma interaction processes (e.g., charge exchange) with the outflowing solar wind (e.g., Sokół et al. 2022; Zirnstein et al. 2022, and references therein). This population in light ions (i.e., hydrogen and helium) was first discovered at 1 AU, upstream of Earth’s bow shock, by the AMPTE/IRM mission (e.g., Möbius et al. 1985) and have since been explored from the inner heliosphere (e.g., Gershman et al. 2013) to beyond the orbit of Pluto (e.g., Randol et al. 2013). Heavier pickup ion species, such as oxygen, nitrogen, and neon, were later discovered at the orbital distance of Jupiter (∼5 AU) by Ulysses (Geiss et al. 1994). Another population of pickup ions are those “planetary PUIs” that originate and escape from a planetary atmosphere and are accelerated via $\overrightarrow{E} \times \overrightarrow{B}$ pickup or other solar wind-induced mechanisms (Jarvinen and Kallio 2014).

To-date, only limited measurements have been made beyond the orbit of Jupiter where a large geometric factor instrument is required to observe hydrogen PUIs, despite the fact that they are more prevalent than in the inner heliosphere due to a reduction in ionization processes (Zirnstein et al. 2022). New Horizons has measured proton and helium pickup ions nearly half way to the termination shock and found that PUI thermal pressure exceeds the thermal solar wind and magnetic pressures in the outer heliosphere by more than an order of magnitude (McComas et al. 2021). Kollmann et al. (2019) added the New Horizons observations to helium PUI measurements made by Cassini from 5 to 10 AU during its cruise to Saturn. They determined that the spectra of suprathermal He^+^ ions with energies of tens of keV from 5-40 AU have spectral indices (i.e., $\gamma $ in $E^{\gamma} $) ranging from -1 to -2. The authors also reported a correlation between high solar wind speeds and soft He+ PUI spectra beyond 10 AU and suggested acceleration at corotating interaction regions (CIRs) and subsequent cooling as a possible explanation. However, these observations have focused primarily on protons and helium PUIs and not heavier elements, such as oxygen, which is shielded from the inner solar system by strong ionization losses (Gloeckler and Geiss 1998).

A mission to Uranus could leverage its long cruise duration and access to the outer solar system to address a multitude of questions surrounding the distribution, evolution, and characteristics of pickup ionization processes from 1 to ∼20 AU. In particular, observations of the thermal, suprathermal, and energetic ion populations with composition determination, especially charge-state where possible, would be critical to enable these investigations. Supplemental observations of the magnetic field and waves would add additional information to help identify both signatures associated with PUIs (e.g., ion cyclotron waves) and transient solar wind structures, such as CIRs and interplanetary shocks, that may accelerate PUIs throughout the heliosphere.

### Interdisciplinary Science

In addition to the targeted space physics science questions outlined in the previous section, PERSEUS would also be able to contribute significantly to several other interdisciplinary science objectives. Kollmann et al. (2020) detail the utility of in-situ particle and field observations to a wide array of non-space physics scientific investigations at both Uranus and Neptune. Below is a brief summary of some of the outstanding topics relevant to Uranus.

#### Planetary Dynamo

A major mystery at Uranus (and Neptune) is how the planet’s dynamo is generated and why it gives rise to its unique multipolar magnetic field (e.g., Soderlund and Stanley 2020). A leading theory suggests that the dynamo may result from the very high electrical conductivity of superionic ices that may be present in the interior of the Ice Giants, though the limited data available makes it hard to test and constrain these models. Multiple orbits of in-situ magnetic field and gravity (via the telecommunications system) measurements from PERSEUS would contribute significantly to addressing this mystery by providing critical measurements to help constrain the higher-order moments of the planet’s intrinsic magnetic field and internal structure. In addition to filling key gaps in understanding of planetary dynamics, learning more about Uranus’s multipolar field may provide insights into the nature of Earth’s magnetic field during field reversals (Caggiano and Paty 2022).

#### Ocean Worlds

Several recent studies suggest that the Uranus system may be host to potential ocean worlds (e.g., Cartwright et al. 2021). PERSEUS’s magnetic field and accompanying plasma measurements would also be important to identify potential ocean world satellites that may exist in the system. Magnetic induction was used to discover subsurface oceans at multiple moons in the Jovian system (Kivelson et al. 1999, 2002). This technique measures the induced magnetic field in a conductive subsurface ocean that results from the varying planetary magnetic field in which the moon exists. The unique configuration of the Uranian magnetic field with its extreme tilt relative to the orbital plane of the moons makes this technique even more effective than at either Jupiter or Saturn because the moons experience much more drastic and periodic variations in the planetary magnetic field. Several recent studies have simulated the potential strength and frequency of the induced signature that may be expected from the Uranian moons as well as the potential implications of those signatures to viable models of their internal structure and composition (e.g., Arridge and Eggington 2021; Cochrane et al. 2021; Weiss et al. 2021).

#### Satellite Interactions with Radiation Environment

The Uranian satellites host some of the darkest surfaces in the solar system (e.g., Cartwright et al. 2021, and references therein). Early work suggested that the darkening of the moon surfaces may be a result of energetic particle interactions with organic ices on the surfaces of the moons (Lanzerotti et al. 1987). Additionally, four (Ariel, Umbriel, Titania, and Oberon) of the five classical moons exhibit stark compositional trends between their leading and trailing hemispheres trends in composition that may at least partially result from charged particle interactions with their trailing hemispheres and/or heliocentric and planetocentric dust impacts with their leading hemispheres (e.g., Cartwright et al. 2015). PERSUES would obtain comprehensive measurements of the energetic particle spectra in the inner magnetosphere necessary to fully characterize the radiation environment encountered by the moons. Furthermore, the suprathermal and energetic ion composition measurements will be critical to determine not only the potential contribution of heavy ion weathering on the moon surfaces, but also the extent to which these moons may be contributing mass to the magnetosphere (Cohen et al. 2023).

#### Ring System

Uranus also hosts dynamically full and apparently haphazard system of dense, narrow rings and small moons (e.g., Showalter 2020; Cohen et al. 2022). Like the classical moons, the small moons and rings are dark and have unknown compositions (Karkoscka 2001). Observations of the rings show that their spectra are flat (de Kleer et al. 2013) and lack the spectral features of H_2_O and CO_2_ ice seen at the classical moons (Grundy et al. 2006); it remains unknown whether the small moons are more similar in their composition to the rings or the larger moons. PERSEUS’s magnetic field and comprehensive (in both energy and composition) charged particle measurements would provide information on the nature and compositional effects of the interaction between magnetospheric particles and the small moons and rings (Cohen et al. 2022).

#### Magnetosphere-Ionosphere-Atmosphere Interactions

Uranus’s thermosphere is mysteriously hot in both the summer and winter hemispheres, which rules out the explanation of solar heating despite the planet’s large axial tilt (Broadfoot et al. 1986; Herbert et al. 1987; Stevens et al. 1993). Unfortunately, which heat transport processes are at work in the middle and upper atmosphere remain unknown as Voyager 2 occultations cannot distinguish between candidate heating mechanisms. Furthermore, it is unclear why Uranus’s upper atmosphere has lower hydrocarbon densities than those observed at any other giant planet (e.g., Melin 2020). PERSEUS’s UV spectrometer would provide additional stellar and solar occultation observations to better constrain the spatial distribution key tracers of vertical transport such as acetylene gas (Cohen et al. 2022). However, the recent results of Turner et al. (2023) may hold additional implications for Uranus’s upper atmospheric temperature. At Earth, global magnetospheric convection is driven by the solar wind (e.g., Dungey 1961). After magnetic fields lines reconnect with the IMF on the dayside the reconnected field lines are dragged anti-sunward along with the solar wind. These magnetic field lines consequently drag the associated ionospheric plasma in the upper atmosphere along with them as the traverse from the dayside to the nightside across the polar caps. During this ionospheric convection, the ionospheric plasma interacts with the neutral particles in the thermosphere via a process known as “Joule heating”, which can have significant effects on processes across the coupled atmosphere-ionosphere-magnetosphere system (e.g., Richmond 2021, and references therein). Given that Turner et al. (2023) concluded that reconnection can occur at some location across the Uranian magnetopause under all seasonal and diurnal configurations, the potential impact of Joule heating may be significant on Uranus’s upper atmosphere. However, the magnitude of the influence of this process has yet to be explored. By addressing its science questions on global magnetospheric transport (Sect. 2.1.1) and the magnetosphere-ionosphere current system (Sect. 2.1.2) PERSUES would also be able to infer how ionospheric convection may be structured and what impacts it may have on heating the atmosphere.

## Mission Concept

### Overview

The PERSEUS mission concept was developed to determine the scope and feasibility of placing a dedicated Heliophysics orbiter into the Uranian system. While the initial trade space included consideration of both an independent mission and a rideshare option as a secondary payload accompanying a future Planetary Science mission, the latter was ultimately abandoned due to feasibility concerns regarding power and communications. For power, a secondary payload would either need its own dedicated power source, which was determined to be unfeasible, or need to only run on batteries, which would drastically limit its lifetime. A direct-to-Earth communication system at Uranus would require significant spacecraft resources and ultimately drive the spacecraft design beyond feasible limitations. Conversely, a relay system relying on the planetary flagship would add significant complexity to the flagship mission communication system by requiring an extra link and the concept of operations as it would need to store and forward both uplink and downlink data for the secondary. As a baseline, the PERSEUS mission design parameters were intentionally addressed using technology currently available or strongly expected to be by the end of the current decade. The PERSEUS concept is presented here at Concept Maturity Level 4 (Wheatcraft and Lewis 2018). A high-level summary of the key mission design parameters to be detailed in the following sections is presented in Table 2.

**Table 2:** A summary of the key flight system element parameters for the PERSEUS mission concept. MEV = maximum expected value; IMU= inertial momentum unit; BOL = beginning of life; EOL = end of life

Flight System Element Parameters	Value and Summary
Design Life	15 years (driven by radio thermoelectric generator (RTG) with 3 years of ground storage)
Mass	913.1 kg (MEV dry), 2035 kg (MEV wet)
Instruments	Plasma Spectrometer, Suprathermal Ion Spectrometer, Energetic Particle Spectrometer, Fluxgate & Search Coil Magnetometers, Plasma Waves Sensor, UV Spectrometer, Education & Public Outreach Camera
**Structure**	
Structures material	Aluminum hexagonal honeycomb
Number of deployed structures	4 (one magnetometer boom and three electric field antennas)
**Thermal Control**	
Type of thermal control	Pump-driven loop using RTG waste heat (similar to Dragonfly)
**Propulsion**	
Estimated Δ*v* budget	1890 m/s capability (1561 m/s required for deterministic burns)
Propulsion type (propellant + oxidizer)	Dual-mode chemical (hydrazine + nitrogen tetroxide (NTO))
Number of thrusters and tanks	12 1-lb thrusters, 1 Leros-1b main engine; 4 tanks (1 fuel, 2 oxidizer)
Specific impulse of each propulsion mode	215 s (mono-prop), 315 s (bi-prop)
**Attitude Control**	
Control method	Spin-stabilized
Control reference	Solar (safe), stars (all other modes)
Attitude control capacity	0.2° (3-*σ*)
Attitude knowledge limit	0.01° (3-*σ*)
Sensor and actuation information	5 coarse sun sensors; two star trackers, one internally-redundant IMU
**Command & Data Handling**	
Flight element housekeeping data rate	<1 kbps
Data storage capacity	265,000 Mbits, utilize Scientist-in-the-Loop (SITL) science operations
Maximum storage record rate	130 kbps
Maximum storage playback rate	5.5 kbps
**Power**	
Power source	1 Mod-1 NG-RTG (16 cores)
Expected power generation	245 W (BOL), 177 W (EOL)
On-orbit average power consumption	22-day orbit: 2 days of science @ 171 W, 5 days of downlink @ 430 W, 15 days of hibernation @ 34 W
Battery type and storage capacity	Li-ion, 420 A-hr, 11.7 kW-hr

### Payload Description

To address the targeted space physics science questions outlined in Table 1, the PERSEUS concept is outfitted with the baseline payload summarized in Table 3 and below; it must be emphasized that the instruments listed in Table 3 are only representative of notional high-heritage instruments that could be used for the proposed mission. Because of the significant propellant mass required to put a spacecraft into orbit at Uranus, the mass efficiency of the entire observatory (i.e., space vehicle subsystems and scientific payload) will be critical to the mission’s feasibility; of course, realistic power and cost limitations were also taken into account. The first task for the proposed study was to assess the resource requirements for each of the instruments versus their performance versus the science objectives outlined in Table 1. It is expected that several of these instruments may require more mass to accommodate additional radiation shielding for the Uranian environment, which has been observed to be very similar to that of Earth (given the limited Voyager 2 observations) and in several instances is harsher than the systems explored by the heritage sensors (see Sect. 3.4.9).

**Table 3:** Summary of the instrument types necessary to achieve the science objectives outlined in Table 1 and potential heritage instruments and trades to be considered in the proposed study. ^*a*^Includes sensors and electronics without Jupiter-specific shielding. ^*b*^Includes CHEMS sensor values plus subset of MIMI suite common electronics. ^*c*^Does not include single 6.3-kg common boom. ^*d*^Includes common DPU and harnessing shared with magnetometers

Instrument Type	Heritage Instrument	CBE Mass (kg)	CBE Power (W)	CBE Survey Data Rate (kbps)	CBE Burst Data Rate (kbps)
Plasma Spectrometer	Juno/JADE-E (McComas et al. 2017)	9.0^*a*^	5.5	0.2	2.6
	Juno/JADE-I (McComas et al. 2017)	11.4^*a*^	6.4	1.4	15.4
Suprathermal Ion Spectrometer	Cassini/MIMI/CHEMS (Krimigis et al. 2004)	9.2^*b*^	9.5^*b*^	1.2	14.0
Energetic Particle Spectrometer	Parker Solar Probe/IS ʘIS/EPI-Lo (McComas et al. 2016)	4.1	5.0	1.0	18.8
Fluxgate & Search Coil Magnetometers	Parker Solar Probe/FIELDS (Bale et al. 2016)	2.2^*c*^	5.2	1.2	15.4
Plasma Waves Sensor	STEREO/WAVES (Bougeret et al. 2008)	13.2^*d*^	15.4	0.6	27.3
UV Spectrometer	New Horizons/ALICE (Stern et al. 2008)	4.4	4.4	n/a	1060
Education & Public Outreach Camera	Juno/JunoCam (Hansen et al. 2017)	3.7	5.9	0.2	n/a
**Total**		**57.2**	**57.3**	**5.8**	**1153.5**

In addition to the instruments included in the baseline payload, the PERSEUS study also considered including an energetic neutral atom (ENA) imager, similar to those that have been used to observe the magnetospheres of Earth and Saturn (e.g., Mitchell et al. 2016, and references therein). However, a preliminary study conducted during the concept development concluded that barring the existence of a clear neutral particle source from a moon, any potential ENA emissions that would yield significant information on the system’s magnetospheric dynamics would be too weak to be detectable by a reasonably-sized instrument. Thus, an ENA imager was excluded from the baseline PERSEUS payload.

#### Plasma Spectrometer

The science objectives for the lower-energy plasma spectrometer require measuring the plasma environment (e.g., energy, angular, and compositional distributions) throughout the Uranian magnetosphere. In particular, current understanding of plasma properties in Uranus’s magnetosphere is limited to observations from the Voyager 2 Plasma Experiment (PLS; e.g., Bridge et al. 1986; McNutt et al. 1987; Sittler et al. 1987; Selesnick and McNutt 1987), which lacked direct compositional measurements and did not cover energies above ∼6 keV (Bridge et al. 1977). It is also important to obtain measurements of the solar wind during the interplanetary cruise and upstream of the bow shock while in orbit at Uranus.

The baseline plasma spectrometer complement for PERSEUS leverages the Juno/JADE instruments (McComas et al. 2017), which would provide sufficient energy, angular, and compositional resolution of the low-energy electron and ion populations, including mass composition, in the Uranian magnetosphere between ∼0.01 and ∼30 keV/q. This would allow for investigation of magnetospheric plasma conditions and configuration, as well as potential radiation belt seed populations.

#### Suprathermal Ion Spectrometer

The science objectives for a suprathermal ion spectrometer require measuring the suprathermal (i.e., tens to hundreds of keV/q) ion population in Uranus’s magnetosphere to distinguish highly-charged species originating from the solar wind from potential singly-charged species picked up from Uranus’s upper atmosphere, rings, and/or satellites and highly-charged species from the solar wind. This population was a severe limitation of the Voyager 2 payload, which lacked ion composition observations in the wide energy range between ∼6 keV/q and 500 keV/nuc (e.g., Mauk et al. 1987; Cohen et al. 2023).

The baseline suprathermal ion spectrometer instrument for PERSEUS assumes the Cassini/CHEMS sensor (Krimigis et al. 2004), which would provide sufficient energy, angular, and compositional (both mass and charge-state) resolution of the suprathermal ion populations from ∼3 to >200 keV/q, thus enabling in-depth investigations of potential ion source populations as well as shock, magnetotail, and radiation belt acceleration processes.

#### Energetic Particle Spectrometer

The science objectives for the energetic particle spectrometer require measuring energy, angular, and compositional distributions of energetic particles in the Uranian system. In particular, current understanding of energetic particles in Uranus’s magnetosphere is limited to observations from the Voyager 2 Low Energy Charged Particle (LECP) instrument (e.g., Krimigis et al. 1986; Cheng et al. 1987; Mauk et al. 1987), which lacked charge-state measurements and only had mass composition determination capabilities at energies above ∼200 keV/nuc (Krimigis et al. 1977); however, damage incurred during the Voyager 2 flyby at Saturn actually raised this threshold to ∼500 keV/nuc (Mauk et al. 1987).

The baseline suprathermal energetic particle spectrometer instrument for PERSEUS assumes the Parker Solar Probe/IS ʘIS/EPI-Lo instrument (McComas et al. 2016; Hill et al. 2017) with appropriate tailoring to enlarge the geometric factor and extend the upper energy range, which would measure electrons and ions (with mass composition) in the Uranian magnetosphere in the range of a 30 keV to >10 MeV/nuc. This would enable investigation of the particle acceleration processes at play in the Uranian radiation belts and throughout the system.

#### Fluxgate Magnetometer

The science objectives for the fluxgate magnetometer require measuring the three-dimensional DC vector magnetic field throughout the Uranian magnetosphere, as well as constraining models of the interior structure of the planet and its moons (and detecting induced fields from potential ocean worlds). In particular, current understanding of Uranus’s magnetic field is limited to observations from the Voyager 2 Magnetic Field Experiment (MAG; e.g., Ness et al. 1986; Connerney et al. 1987; Voigt et al. 1987), which comprised two systems of paired fluxgate magnetometer sensors on an extended boom (Behannon et al. 1977).

The baseline fluxgate magnetometer instrument for PERSEUS assumes the Parker Solar Probe/Fields/MAG sensor (Bale et al. 2016), which would measure DC magnetic fields up to ∼140 Hz over a dynamic range of ±65,000 nT. As implemented on Parker Solar Probe, PERSEUS would carry two sensors mounted on a shared extended boom in a gradiometer configuration (Russell et al. 2013) to help characterize and remove electromagnetic interference from the spacecraft. This would enable investigation of the magnetospheric topology, as well as supply invaluable measurements for the calculation of particle pitch angles.

#### Search Coil Magnetometer

The science objectives for the search coil magnetometer require measuring the higher-frequency three-dimensional vector magnetic field and waves environment at Uranus. Unfortunately, the Voyager 2 payload had a significant frequency gap that included ion-mode electromagnetic and electrostatic waves between the MAG instrument and the Plasma Wave System (PWS; Scarf and Gurnett 1977). Including a triaxial search coil magnetometer sensor on PERSEUS would be important to address this largely unexplored frequency regime at Uranus.

The baseline search coil magnetometer instrument for PERSEUS assumes the Parker Solar Probe/Fields/SCM sensor (Bale et al. 2016), which would measure three components of the AC magnetic field from 10 Hz to 50 kHz and a single component from 1 kHz to 1 MHz. Like Parker Solar Probe, PERSEUS would accommodate the search coil magnetometer on the same boom as the fluxgate magnetometer sensors. This would enable investigation of ion-mode waves that may exist in the system and the potential particle-wave interactions that may arise from them.

#### Plasma Waves Sensor

The science objectives for the plasma waves sensor require measuring the plasma wave power and spectra throughout the Uranian magnetosphere. In particular, current understanding of waves in Uranus’s magnetosphere is limited to observations from the aforementioned Voyager 2/PWS (e.g., Gurnett et al. 1986; Kurth et al. 1987; Scarf et al. 1987), which included two 10-meter antennae shared with the planetary radio astronomy experiment (Scarf and Gurnett 1977).

The baseline plasma waves instrument for PERSEUS assumes the STEREO/WAVES sensor (Bale et al. 2008; Bougeret et al. 2008), which would measure three components of the electrostatic and electromagnetic waves from 10 kHz to 16 MHz. Like STEREO, PERSEUS would accommodate the with a set of three 6-m orthogonal electric monopole “stacer” antennas.

#### Ultraviolet Spectrometer

The science objectives for the UV spectrometer require measuring the H_2_ bands (80-160 nm) and Lyman-$\alpha $ (121.6 nm) emission bands in the magnetic polar regions of the planet. In particular, current understanding of the Uranian aurora come from limited remote observations by HST (e.g., Lamy 2020) and in-system observations from Voyager 2/UVS (e.g., Gurnett et al. 1986; Kurth et al. 1987; Scarf et al. 1987), which covered the wavelength range of 50-170 nm with 1-nm resolution (Broadfoot et al. 1977).

The baseline plasma waves instrument for PERSEUS assumes the New Horizons/Alice sensor (Stern et al. 2008), which covers a spectral passband from 52–187 nm with a 0.3–0.6-nm spectral point spread function across a 6°-long field-of-view. This would enable investigations of the auroral precipitation and magnetosphere-ionosphere coupling mechanisms at play at Uranus.

### Mission Design

**Fig. 1:**
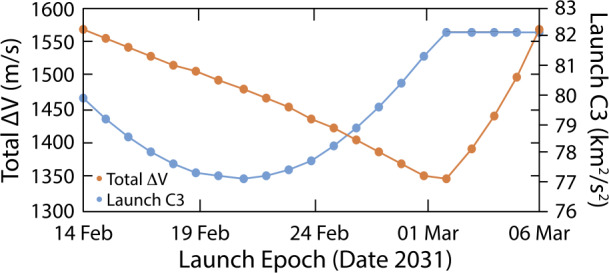
PERSEUS 2031 primary launch period launch C3 and total deterministic $\Delta v$ through science orbit insertion

**Fig. 2:**
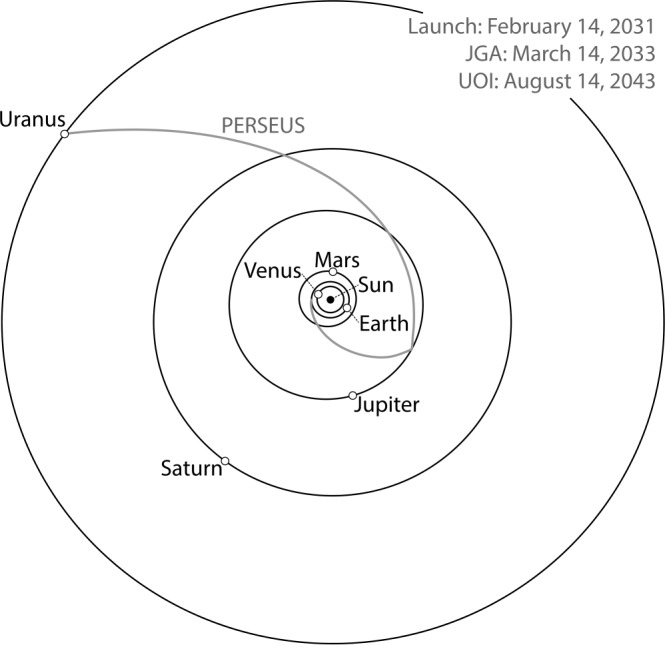
Baseline 2031 EJU interplanetary trajectory for PERSEUS viewed from ecliptic north

The PERSEUS mission design utilizes a high-energy launch injection with a C3 that varies from 77.15 to 82.0 km^2^/s^2^ (Fig. 1). The primary 21-day launch period is 14 February 2031 to 6 March 2031. The worst-case launch period deterministic $\Delta $*v* of 1,561 m/s includes deep-space maneuvers (DSMs), the Uranus orbit insertion (UOI) maneuver (modeled as a finite-burn), as well as the Uranus science orbit period reduction burn. The favorable relative positioning of Jupiter and Uranus during the 2031/2032 timeframe enables a Jupiter flyby approximately two years after launch (March 2033), and subsequent capture in the Uranus system 12.5 years after launch (Fig. 2). The orbit insertion sequence is nearly identical across the launch period, and the Uranus arrival date can be adjusted to target the same capture conditions across the launch period. The deterministic maneuver between Earth and Jupiter accounts for the majority of the variation in $\Delta $*v* across the launch period.

All trajectories were modeled in high-fidelity using the Evolutionary Mission Trajectory Generator (EMTG), a variable fidelity trajectory optimization software package used by the Johns Hopkins University Applied Physics Laboratory (APL) (Ellison et al. 2018a, 2018b). The EMTG tool has been used to support several NASA missions and concepts from initial development (e.g., DAVINCI, Dragonfly, and Europa Clipper) through operations (e.g., Lucy and OSIRIS-Rex) (Englander et al. 2019). Point-mass gravity from Mercury, Venus, Earth/Moon, as well as system gravity from Mars, Jupiter, Saturn, Neptune, and Pluto is modeled throughout the trajectory. While inside the spheres of influence of Earth, Jupiter, or Uranus, gravity harmonics were also modeled. Earth gravity harmonics were computed using an 8×8 GIF48 model (Ries et al. 2011), Jupiter gravity harmonics were computed using a 6×0 JUP230 model (Jacobson 2010), and Uranus gravity harmonics were computed using a 4×0 URA083 model (Jacobson 2007).

The Jupiter gravity assist (JGA) has a closest approach altitude of approximately 67,600 km (∼2 R_J_), which is inside the orbit of Amalthea. The closest approach altitude increases across the launch period and is over 85,000-km (2.2-R_J_) altitude for launch close. The flyby is inclined at 136° relative to Jupiter’s equator, and avoids the highest-radiation regions of the Jovian inner magnetosphere.

The baseline UOI maneuver occurs near periapsis at ∼1.06 R_U_ on 14 August 2043. The insertion maneuver is executed using the Leros-1b main engine and has a duration of 47.8 min imparting a finite-burn $\Delta $*v* of 1,165 m/s. The insertion maneuver requires a slow attitude slew such that the thrust acceleration vector approximately tracks the spacecraft anti-velocity direction.

**Fig. 3:**
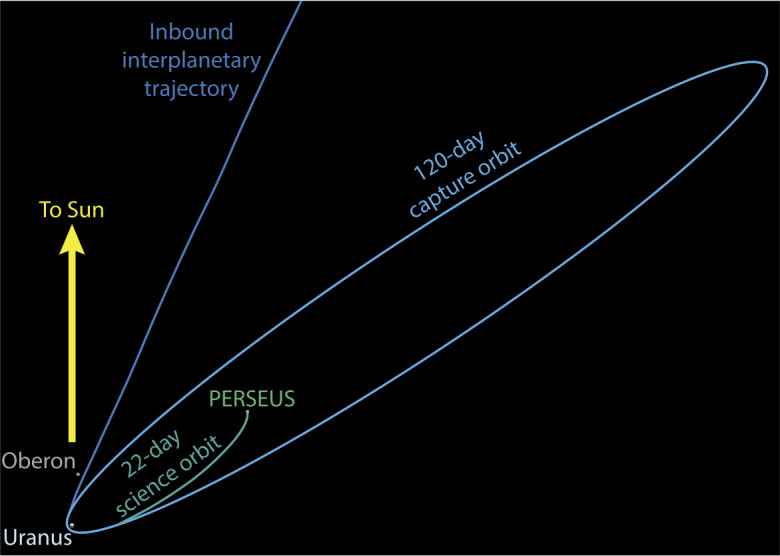
PERSEUS captures into a 120-day science orbit before reducing its orbital period to 22 days. The capture trajectory is shown as viewed from ecliptic north with the Sun-Uranus vector shown in yellow

**Fig. 4:**
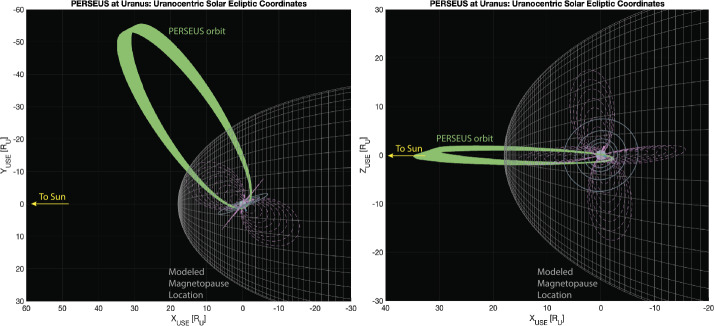
The PERSEUS orbit is high-inclination relative to the planet’s poles, but remains low-inclination relative to the ecliptic plane. PERSUES’s apoapsis is in the dawn dayside sector and precesses slightly towards to the Sun-Uranus line (as viewed from ecliptic north) over the two-year baseline mission. This simulation shows the PERSUES orbit (green) relative to an idealistic magnetopause (gray mesh surface) based on the Voyager 2 observations, the orbits of several inner moons (blue), and several radial distances and MLTs of dipolar magnetic field lines (dashed pink)

The UOI maneuver places the spacecraft into a 120-day capture orbit. A 125-m/s period reduction maneuver is performed at the periapse of the capture orbit, which targets the final science orbit with a period of approximately 22 days (Fig. 3). The 22-day science orbit is stable over the primary science mission, with the periapse altitude experiencing a secular increase over time. Uranus’s gravity harmonics cause the science orbit to precess towards the Sun-Uranus vector over the course of the primary science mission (Fig. 4). However, it should be noted that the only in-situ gravity data available for the Uranian system is from the Voyager 2 flyby and is severely limited. Given this lack of gravity field knowledge, the science orbit trajectory has uncertainty that can only be refined once the spacecraft is in orbit around Uranus and orbit determination can be performed.

The PERSEUS trajectory features a backup opportunity approximately fifteen (15) months later from 1 May 2032 to 21 May 21 2032. This launch period utilizes the same Earth-Jupiter-Uranus (EJU) sequence, with a time of flight of 13.33 years from launch until Uranus system capture. The worst-case deterministic $\Delta $V across this launch period is 1,460 m/s, which is ∼6% less than that for the baseline 2031 launch window. The backup launch period was modeled at the same level of fidelity as the primary lauch period.

**Fig. 5:**
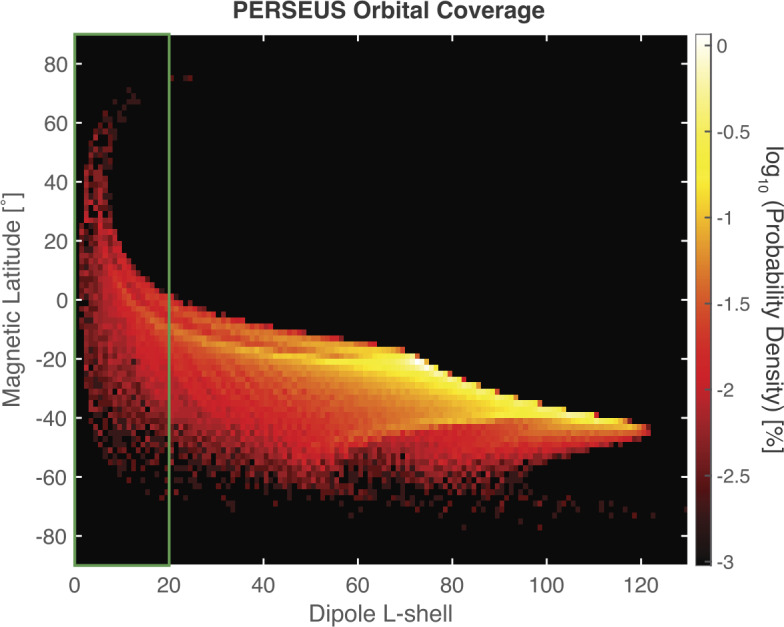
The PERSEUS mission design takes advantage of Uranus’s uniquely large obliquity and dipole tilt to access a large range of magnetic latitudes – specifically inside the nominal distance of the magnetopause (green box) – from an orbit at low inclination relative to the ecliptic plane

Using the baseline 2031 EJU trajectory, PERSEUS would arrive at Uranus in mid-2043 and explore the system just before equinox, a very different configuration from the solstice configuration encountered by Voyager 2 (Stone and Miner 1986). It must also be noted that the ability to sample the magnetotail and target nightside magnetospheric dynamics is limited by the realities of the interplanetary trajectory and spacecraft capabilities. These together combine to constrain the spacecraft line of apsides to within ∼50° of the Sun-Uranus line on the dayside (Fig. 4). Unfortunately, the long period of precession keeps the PERSEUS apoapsis relatively close to 08:00 local time (LT) for the duration of the two-year baseline prime mission. Despite this, PERSEUS takes advantage of the dramatic variability of the Uranian magnetosphere to provide significant magnetic latitude (MLAT) and radial (i.e., L-shell) coverage over the prime mission from a relatively fixed orbit in the ecliptic plane (Fig. 5). From its baseline 22-day science orbit with apoapsis in the pre-noon local time region, PERSEUS is able to repeatedly sample the dayside magnetopause and bow shock as well as the inner magnetosphere/radiation belts, magnetotail regions (at low altitudes), and over the magnetic poles.

**Fig. 6:**
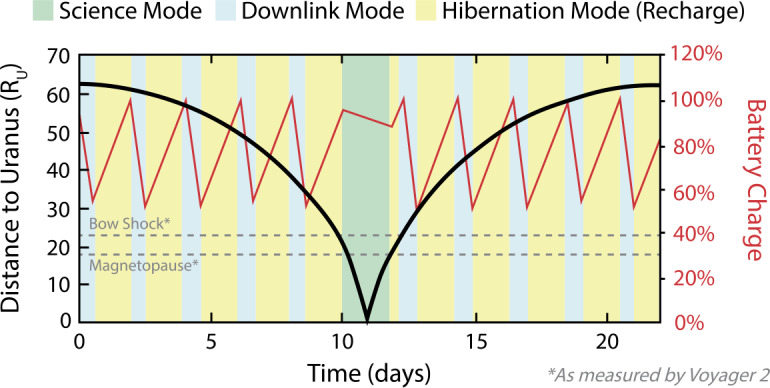
The PERSEUS spacecraft and CONOPS are designed to rely upon significant periods in each orbit in hibernation mode interspersed between periods of data downlink. Science is primarily captured around periapsis while PERSEUS is in the Uranian magnetosphere

The PERSEUS concept of operations (CONOPS) is driven largely by the power subsystem detailed in Sect. 3.4.1. The PERSEUS spacecraft borrows heavily from the spacecraft architecture and CONOPS used on the Dragonfly mission, which utilizes a passive cooling loop that leverages waste heat from only a single NG-RTG power supply with a significant (2100-Whr) battery; for comparison, recently-considered flagship-class Planetary Science missions have required four or more Enhanced Multi-Mission RTGs (eMMRTGs) (Hofstadter et al. 2017) or several NG-RTGs (Simon et al. 2022). Figure 6 presents an overview of the varying modes of the PERSEUS spacecraft throughout a typical 22-day (1.2 R$_{\mathrm{U}}\ \times $ 62 R_U_) science orbit relative to the distance from Uranus and the battery’s charge. This approach allows for ∼1.8 days of science collection inside of the magnetopause (observed at 18 R_U_ by Voyager 2 (Ness et al. 1986)) per 22-day orbit; this essentially captures the same duration of in-situ data as Voyager 2 flyby every month for the entire baseline two-year prime mission. Approximately 15% of each 22-day orbit is spent downlinking science data to Earth. The current best estimate (CBE) data rate for the payload summarized in Sect. 3.2 is 11.33 kbps – assuming 1% (or ∼1 hr) burst-mode collection per orbit; this totals 1.85 GB per orbit.

Given the limitations on the downlink that are assumed relative to the data that can and likely will be collected in orbit, PERSEUS will implement a proven Scientist-in-the-Loop (SITL) operational architecture similar to that implemented on missions like Magnetospheric Multiscale (MMS) (e.g., Baker et al. 2016; Fuselier et al. 2016). In this process, trained members of the science team regularly comb through lower-resolution data to identify events of the highest scientific interest for priority higher-resolution data downlink. As outlined by Argall et al. (2020), this process has also informed a more sophisticated automated selection leveraging a machine learning model process that could also potentially be applied.

### Flight System

The point design presented in this section enables the mission to meet all science requirements with a high-heritage, low-power, cost-conscious bus design. PERSEUS employs a hibernation-heavy CONOPS (Fig. 6) to reduce the average power draw of the flight system. Passive spin-stabilization allows the flight system to power off all attitude control system (ACS) components and most avionics components while in hibernation. Additionally, a pump-driven thermal loop, utilizing RTG waste heat and passively-controlled radiators, keeps all components within operational temperature limits without additional heater power. While hibernating, the spacecraft is limited to a receive-only mode that recharges the battery, runs the thermal loop, and provides rudimentary fault detection for situations requiring a time-sensitive response. At Uranus this hibernation state is the only power-positive flight system state. Table 4 summarizes the mass and power allocations of the PERSEUS flight system by subsystem.

**Table 4:** Mass and power (for different operational modes) allocations for PERSEUS

	Mass			Operational Power Modes								
				Science			Downlink			Hibernation		
Subsystem	CBE (kg)	Cont.	MEV (kg)	CBE (W)	Cont.	MEV (W)	CBE (W)	Cont.	MEV (W)	CBE (W)	Cont.	MEV (W)
Science Payload	64.3	10%	70.8	54.0	30%	70.1	-	-	-	-	-	-
Mechanical (parametric)	241.1	15%	277.2	-	-	-	-	-	-	-	-	-
Propulsion	108.5	3%	111.8	-	-	-	-	-	-	-	-	-
Avionics	8.1	15%	9.3	25.0	15%	28.8	25.0	15%	28.8	-	-	-
Power	176.4	13%	199.7	32.1	17%	37.5	32.1	17%	37.5	15.8	19%	18.8
Attitude Control System	18.3	10%	20.1	8.6	5%	9.0	10.7	5%	11.2	-	-	-
Thermal	85.2	7%	90.9	13.0	10%	14.3	13.0	10%	14.3	13.0	10%	14.3
Communications	55.6	13%	62.5	5.9	15%	6.7	309.5	5%	325.9	-	-	-
Harness (Parametric)	61.5	15%	70.7	4.2	20%	5.0	11.7	7%	12.5	0.9	15%	1.0
**Flight System Dry Mass**	**818.9**	**11%**	**913.1**									
**Flight System Power Loads**				**142.6**	**20%**	**171.4**	**402.0**	**7%**	**430.2**	**29.7**	**15%**	**34.1**

**Fig. 7:**
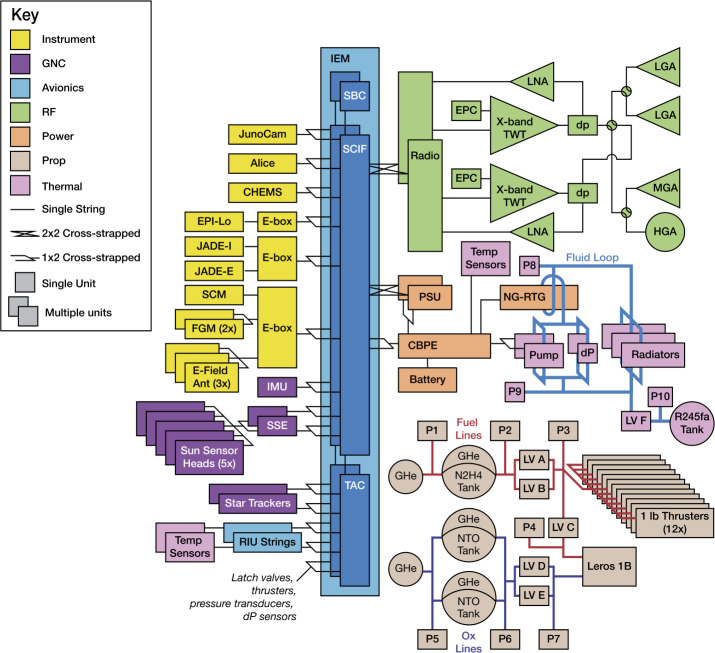
Block diagram of the PERSEUS flight system

All operations conducted outside of hibernation — including all science collection, downlink, and thruster firings — rely on discharging the battery to meet instantaneous power requirements. A similar CONOPS is used on the Mars rovers and is planned for the Dragonfly mission to Titan (Lorenz et al. 2018). The baseline spacecraft design prioritizes cost-effective, reliable, and flight-proven design solutions with no technology development required. A block diagram of this point design is presented in Fig. 7.

For the baseline architecture outlined here, it is assumed that the spacecraft will largely be in hibernation during the extended cruise to Uranus and will break that once every six months for observatory checkout, as New Horizons (Fountain et al. 2008) did. However, the hibernation-heavy CONOPS for PERSEUS is largely driven by the limited power available in-orbit at Uranus and the degrading power supply of the RTG near the end of the mission lifetime. Therefore, the cruise CONOPS could be tailored to allow multiple instruments to stay on for extended periods to achieve heliospheric science leveraging the weekly contacts with NASA’s Deep Space Network (DSN) already baselined during the interplanetary cruise.

#### Electrical Power

The power generation, storage and distribution system for PERSEUS is designed around the use of a single Mod-1 NG-RTG as its power source. RTGs are the only viable, high-TRL power generation option at Uranus and the Mod-1 NG-RTG offers the most power for a given amount of Pu-238 fuel. A solution with two Multi-Mission RTGs (MMRTGs) was also considered, but delivered less than half the data volume return per orbit. For the PERSEUS point design, the Mod-1 NG-RTG is assumed to produce 245 W at fueling with a power degradation rate of 1.9%. At the end of the science phase, 17 years after fueling (including three years of ground storage before launch), the Mod-1 NG-RTG is assumed to produce 177 W (Zakrasjek 2021).

**Fig. 8:**
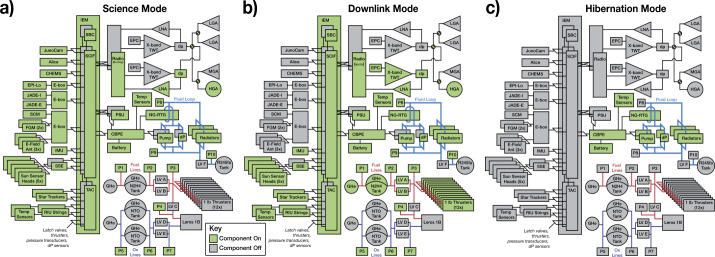
Block diagrams of the PERSEUS flight system highlighting which elements are powered in each operating mode. Note that the PERSEUS spacecraft design and CONOPS relies on a hibernation mode where most spacecraft elements are powered off

PERSEUS utilizes a single, 28-V bus architecture. The internally-redundant Critical Bus Power Electronics (CBPE) handles RTG shunt regulation, battery charging, battery cell balancing, rudimentary fault detection, and power switching for components powered during hibernation and rudimentary fault detection. When not hibernating, the block-redundant Power Switching Unit (PSU) handles load switching for the majority of the flight system loads. Both the CPBE and PSU are based heavily on Dragonfly designs, which leverage significant heritage from electronics used on past APL missions. The PERSEUS CPBEs are tailored to work with the higher power output of the NG-RTG (as opposed to the MMRTG) and a lower-voltage battery configuration. A large, 420-Ahr (11.7-KWh) battery utilizes twenty-four GS Yuasa LSE134 cells in a 3S8P configuration. The battery is constructed using the same techniques proven on several previous missions, including the Van Allen Probes (Butler 2018). A charge rate of C/100 is possible at Uranus while in hibernation mode. Assuming a maximum depth of discharge of 50%, this architecture enables about forty-five (45) hours of continuous science collection per science orbit and 128 hours of total downlink time divided between ten contacts per orbit. Figure 6 presents a notional look at the expected discharge cycles for a single orbit. Figure 8 summarizes which components are powered in each of the three primary operational modes: science, downlink, and hibernation.

#### Communications

The X-band radio frequency (RF) communications system design is driven primarily by the UOI maneuver coverage and science data return. UOI is completed as a single burn with a large bi-propellant engine that is mounted on the spacecraft spin-axis. As the hour-long burn progresses, the vector to Earth migrates in the body frame necessitating the use of a toroidal beam antenna to maintain communication. Two, block-redundant 300-W (170-W RF) COTS traveling wave tube amplifiers (TWTA) combined with the toroidal beam antenna enable an X-band beacon during UOI. While at Uranus, the large Earth range necessitates the use of a high gain antenna (HGA) for science data return. The same 3.1-m HGA used for Europa Clipper (Bray 2020) was selected for its design heritage. This system enables a science data return of 5.5 kbps at Uranus-Earth ranges while only requiring a pointing accuracy of 0.2° (assumes one 34-m DSN antenna). A similar Ka-band architecture would be much more difficult to achieve with a spin-stabilized spacecraft as it requires 0.06° of pointing accuracy. Two low gain antennas (LGAs) mounted on opposing decks enable 4$\pi $-steradian coverage for launch, detumble and early cruise and safe-mode operations. The HGA is used for safe-mode operations in late cruise and at Uranus. Two cross-strapped, high-heritage Frontier Radios – developed by APL – include X-band transmit and receive capability (e.g., Haskins et al. 2016). Each radio also includes an evacuated miniature crystal oscillator (EMXO) providing primary clocking for the entire spacecraft. Only one amplifier is powered at a time. Compensation magnets are placed around both TWTAs, based on magnetic characterization testing, to enable the magnetic field measurements required by the science objectives.

#### Thermal

The PERSEUS thermal design utilizes a pump-driven thermal loop to keep all the components, including the RTG, in operational temperatures while minimizing power requirements. The NG-RTG continuously dissipates between 3000 and 4000 W of waste heat throughout the mission (Zakrasjek 2021). Other nuclear-powered interplanetary spacecraft, like New Horizons (Fountain et al. 2008), have radiated a majority of the excess waste heat into space. PERSEUS plans to capture some of this waste heat and pump it back into the flight system to alleviate the need for additional operational and survival heaters. The fluid loop pumps R245fa refrigerant into an a high-emissivity absorber mounted near the base of the RTG. The absorber is designed to capture ∼10% of the RTG waste heat. The heated fluid is then pumped around the flight system to keep all components within operational limits including the propulsion system, spacecraft panels, thruster valves, and battery. Included in the loop are high-emissivity radiators that passively control the amount of heat they dissipate via temperature-controlled valves as used on Europa Clipper. Two pumps, which are never operated simultaneously, keep the fluid moving through the system in all modes. Standard spacecraft thermal design processes are maintained outside of the thermal loop. Radiative losses are constrained by encasing the majority of the flight system in multi-layer insulation (MLI) and thermally isolating the large-aperture HGA.

#### Propulsion

A dual-mode, pressure-regulated propulsion system utilizes high-heritage components to provide spin control, attitude control, and the required system $\Delta $*v* with high-heritage components utilizing hydrazine (N_2_H_4_) and nitrogen tetroxide (N_2_O_4_). The system consists of one 645-N bipropellant main engine (Leros-1b), twelve 4.4-N monopropellant thrusters, and the components required to control the flow of propellant and monitor system health and performance. A bipropellant mode is required to meet the $\Delta $*v* requirements while remaining the launch vehicle mass delivery capability. The main engine is mounted on the spin axis to enable spinning during the large UOI and apoapsis lowering maneuvers. A similar concept of operations was proven on Juno (Bolton et al. 2017). One large fuel tank is mounted inside the spacecraft central cylinder with two oxidizer tanks mounted in opposing bays. Two helium pressurant tanks are also mounted in opposing bays to main spin-balance throughout the mission.

#### Mechanical

**Fig. 9:**
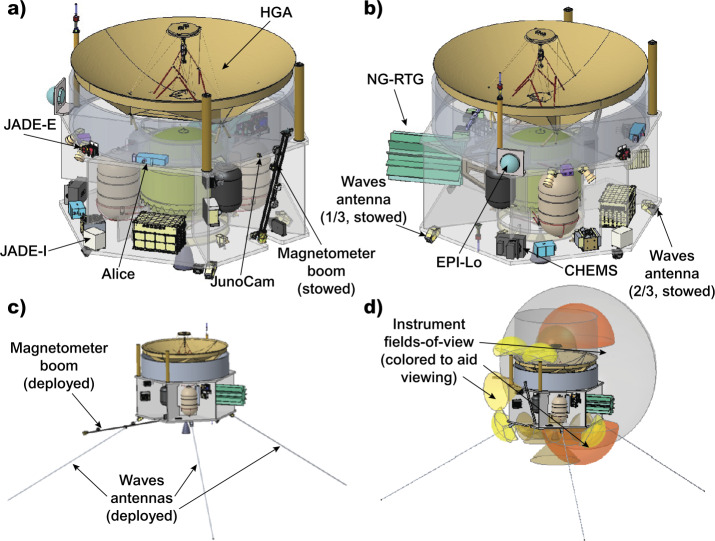
Views of the PERSEUS spacecraft in its stowed (a-b) and deployed (c) configurations. The spacecraft is able to accommodate all of the instrument fields-of-view (d)

The PERSEUS mechanical design accommodates the instruments and flight system components in a compact, mass-efficient, and spin-capable design. The current layout provides clear fields-of-view (FOVs) for all instruments (Fig. 9d), thrusters, star trackers, RTGs, thermal loop radiators, and Sun sensors. The mechanical configuration for PERSEUS is shown in Fig. 9 in both the stowed (Fig. 9a-b) and deployed (Fig. 9c) states.

The primary structure is composed of aluminum honeycomb and consists of a central cylinder, two hexagonal decks, and six subpanels. The hexagonal decks are mounted on either end of the central cylinder with the subpanels radially distributed around the central cylinder to form six bays. A large fuel tank is mounted inside the central cylinder with two oxidizer tanks mounted in opposing bays. Flight system electronics are mounted directly to the primary structure. Instruments are mounted using secondary structure brackets to accommodate FOVs and pointing requirements. The clamp-band launch vehicle adapter is mounted to the central cylinder to provide a direct load path during launch. PERSEUS uses a standard 47-in clamp-band suitable for flight with the baselined Falcon Heavy Expendable launch vehicle. The main engine is recessed inside the clamp-band while the 3.1-m HGA is mounted on the opposite side of the cylinder. The RTG is mounted to the central cylinder via secondary structure. The magnetometer boom is deployed through the use of flight-qualified frangibolts.

The PERSEUS mechanical design is comparable to that of Van Allen Probes (Kirby et al. 2013), maximizing packaging and structural efficiency while ensuring a central center of gravity and proper inertias for a spin-stabilized flight system. Although compact, the structural design allows for significant flexibility in the movement of boxes for spin balancing.

#### Attitude Control System

The PERSEUS attitude control system (ACS) enables the spin-stabilized flight system to meet all performance requirements set by the payload, navigation, communication, and propulsion elements. All ACS components would be competitively selected, commercially-available, off-the-shelf (COTS) components.

The spacecraft is dynamically spin-stable (i.e., is a major-axis spinner) and, therefore, needs very little active control. For the entire cruise and science phase, the ACS keeps the spacecraft in a simple spin that can be adjusted by firing the twelve, 1-N ACS thrusters. The spacecraft precesses as needed to enable Earth communication and perform maneuvers. Statistical trajectory correction maneuvers (TCMs) are performed in any direction with the 1-N thrusters. Spin-stabilization is maintained throughout the UOI performed on the Leros-1b engine. Throughout the mission the spacecraft would evaluate its spin-axis orientation after every hibernation period and readjust if needed in a concept of operations similar to the New Horizons “tweak-ups” (Fountain et al. 2008). The star tracker system provides nominal attitude determination when the spacecraft is outside of the hibernation mode. The star tracker optical heads are aligned to minimize concurrent disruption by the thruster plumes, spacecraft structure, the Sun, or other celestial bodies. The IMU provides spacecraft rotational rate and linear acceleration information for all TCMs and “tweak-ups” but is otherwise powered off. Sun sensors are used in some safe modes to establish a Sun vector to aid in establishing communication with Earth. To aid in mass, power and cost estimation for this concept, the Sodern Hydra star tracker system, Northrup Grumman Scalable SIRU, and Redwire digital solar aspect detectors (DSADs) are assumed as representative high-heritage solutions.

#### Avionics

An internally-redundant Integrated Electronics Module (IEM) and two block-redundant strings of eight remote interface units (RIU) contains all the hardware essential to run onboard flight software (FSW), provide data interfaces for all components, store science and engineering data, provide thruster control, and provide a sync pulse for power supply switching frequency synchronization.

The IEM is internally block-redundant with two, independent strings of core avionics components. Each avionics string consists of one single board computer (SBC), two spacecraft interface (SCIF) cards, one thruster/actuator controller (TAC) and one DC/DC converter. The SBC includes a single radiation-hardened, UTC-700 100-MHz processor with 256 MB of SDRAM, 8 MB of MRAM for code storage, and 256 GB of flash storage for science and engineering data storage. The SCIFs are responsible for interfacing with the majority of the spacecraft components via SpaceWire, UART, or I2C interfaces. One SCIF SpaceWire router distributes virtual time codes throughout the system using the radio’s precision oscillator as its clock source. The primary SCIF also provides a sync pulse to the power supply switching frequencies across the spacecraft to minimize interference with the electric field instruments suite. This technique has been demonstrated on multiple missions, most recently Parker Solar Probe (Fox et al. 2016). The Redundancy Controller Card (RCC) handles core avionics string selection and cross-strapped component selection similar to Parker Solar Probe. The RIUs provide distributed, multi-channel data acquisition for temperature sensors and other housekeeping data.

#### Flight Software

The science data storage and downlink are the primary, mission-specific drivers of the PERSEUS FSW design. FSW uses a layered architecture to encapsulate functionality into multiple, distinct applications. Limited new application development is required for PERSEUS due to substantial re-use from past missions. AES-128 encryption, currently being developed for the Interstellar Mapping and Acceleration Probe (IMAP) mission (McComas et al. 2018), is baselined to comply with NASA’s new uplink encryption guidelines. The FSW includes a rule-based autonomy system that monitors system health outside of hibernation.

#### Radiation Environment

**Fig. 10:**
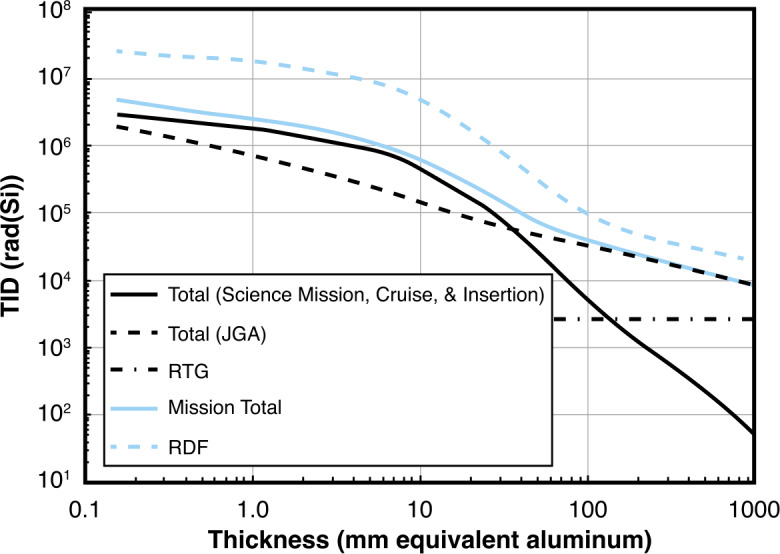
Preliminary analysis of the expected TID for the PERSEUS mission including contributions from the solar protons during cruise, as well as energetic particles trapped at Jupiter and Uranus

Preliminary analysis of the anticipated Total Ionizing Dose (TID) for PERSEUS revealed that 100-krad parts are acceptable given radiation shielding equivalent to 100 mm of aluminum. The TID estimate includes contributions from solar protons during cruise (radiation design factor (RDF) of 2×), trapped protons and electrons at Jupiter during JGA (RDF of 2×), and trapped protons and electrons at Uranus during UOI and the baseline science phase (RDF of 10×) (Fig. 10). Jupiter fluxes and fluences were calculated using the JPL GIRE model (Garrett et al. 2016, 2017). Comparisons with other models (e.g., Natural Space Environments Tools (NSET) and GRID (e.g., Martinez-Sierra 2017; Martinez-Sierra et al. 2021)) were favorable giving high confidence to these results and a lower RDF. Uranus fluxes/fluences were calculated via the JPL Uranian Radiation Model (UMOD) (Garrett et al. 2015). This model only encompasses about 5% of the PERSUES science orbit, does not extend below 4.5 R_U_ (∼1.4% of the science mission is spent below this limit), and is based on a limited set of data gathered from Voyager 2 Cosmic-Ray Detector System (CRS) (Stone et al. 1977) and LECP (Krimigis et al. 1977) observations. Recent comparisons of UMOD with Voyager 2 data were within a factor of 2-5× (Jun et al. 2019). Given the uncertainties in the Uranus model a higher RDF was assigned for this contribution.

### Mission Operations

Ground support for PERSEUS, summarized in Table 5, is provided by the DSN. A 34-m beam-waveguide DSN antenna is assumed for all links except UOI, which requires either a 70-m antenna or an equivalent array of 34-m antennas. Throughout the mission, data rates are artificially capped to those available at Uranus ranges to reduce the number of unique configurations and simplify operations. Although the observatory is not capable of supporting continuous coverage, continuous coverage is still requested around unique events to account for off-nominal approach and departure conditions.

**Table 5:** Summary of the ground support planned for various phases of the PERSUES mission concept

Phase	Coverage Cadence	Phase Duration (days)	Antenna	Downlink Rate (kbps)	Data Volume (Gb)
Launch	Continuous	7	LGA or Torodial	7.8	4.7
Commissioning	8-hr tracks daily	60	LGA or Torodial	7.8	13.5
Inner Cruise	One 8-hr track weekly	701	LGA, Torodial or HGA	7.8	22.5
Jupiter Flyby	Continuous	14	HGA	7.8	9.4
Outer Cruise	One 4-hr track weekly	3791	HGA	5.5	42.9
UOI	Continuous	14	Torodial during burn,HGA outside burn	tones (in-burn),5.5 (on HGA)	N/A
Uranus Coast	One 4-hr track per day*	110	HGA	5.5	8.7
Period Reduction Maneuver	Continuous	14	Torodial during burn,HGA outside burn	tones (in-burn),5.5 (on HGA)	N/A
Safe Mode	As needed	N/A	HGA	7.8125 bps	N/A
Science Phase	One 4-hr track daily*	730	HGA	5.5	57.8
*Average daily downlink based on science concept of operations					

Fault detection on PERSEUS is accomplished via a combination of rule-based autonomy when outside of hibernation and a simple FPGA-based critical telemetry monitoring system that resides in the CBPE during hibernation (see Sect. 3.4.1). The system responds to time-sensitive critical faults by initiating a safe-mode sequence designed to give the system the best chance at recovery. The sequence includes 1) safely terminating current activities; 2) transitioning to a communication capable state with a beacon enabled; and 3) if needed, autonomously performing a spin-axis repositioning maneuver. The LGAs and toroidal beam antenna are used to close the safe-mode link out to Jupiter. After the Jupiter flyby only the HGA is used. Configurable autonomy parameters manage which antenna is selected as part of the safe-mode sequence. Sun sensors provide full 4$\pi $-steradian coverage throughout the mission enabling the spacecraft to always find the Sun. The autonomous repointing sequence repositions the observatory spin axis toward the Sun before the Jupiter flyby and toward the Earth after the flyby. If Earth knowledge is unknown, then the observatory enters an Earth-acquisition mode that sweeps the HGA in a circle around the Sun. The radius of the circle is stored in configurable parameters managed by the mission operations team. The safe-mode sequence, estimated to take about thirty minutes, is not power positive.

After completing the sequence, the beacon is continuously broadcast until the battery state-of-charge drops below a programmable threshold, at which point the system transitions to hibernation mode. The observatory stays in hibernation mode until either the battery state-of-charge raises above a programmable threshold or the system receives the dedicated “wake-up” command. All subsequent transitions out of hibernation mode begin with another execution of the safe-mode sequence until the observatory is restored to the nominal operations cycle by the mission operations team. The safe mode sequence is disabled during UOI.

### Key Trades

Power consumption and overall system complexity were the primary drivers for most trades in the formulation of the PERSEUS mission concept. In general, lower average power and simpler systems were also evaluated to be cheaper and less risky. Nuclear-powered RTGs are the only high-TRL option for powering a spacecraft at Uranus. RTGs are not only costly to both procure and accommodate, but there are also availability concerns given the dwindling radioisotope fuel (Pu-238) stockpile and limited production rates (NASEM 2022). These concerns drove PERSEUS to prioritize solutions that minimize the average power required during the science phase and thus minimize the amount of radioisotope fuel required.

A Class-B risk classification, as defined by NASA Procedural Requirements (NPR) 8705.4A Appendix C (NASA 2021a), is assumed due to the lifetime, complexity, and estimated life-cycle cost of PERSEUS. This risk classification assumption drives PERSEUS to a redundant spacecraft bus design with limited single-point failures. Simple flight system design reduces the mass and cost impacts caused by the redundancy requirement. The PERSEUS design prioritizes solutions with the minimal amount of unique hardware required. Table 6 presents the key trades explored for the PERSEUS concept as well as the selections and associated rationale for each.

**Table 6:** Key trades explored during the development of the baseline PERSEUS mission design concept

Trade	Options Evaluated (Selection in bold)	Rationale
UOI Method	**Insertion burn** *vs*. aerocapture	• New launch vehicles enable trajectories with insertion burn magnitudes comparable to operating missions and total flight times within the 17-yr estimated RTG lifetime (assumes 3 year ground storage) • Insertion burns are much higher TRL, especially given the uncertainty in the Uranian atmospheric composition.
ACS Control Method	**Spin-stabilization** *vs*. dual-mode ACS system (spin and three-axis capable)	• Payload requires at least one spin-stabilized flight system mode for science data collection. • Three-axis control modes require more power. Three-axis control achieved via thrusters requires constant power for the catalyst bed heaters. Three-axis control via reaction wheels requires additional components and more power. • All mission objectives can be completed with a solely spin-stabilized flight system.
RF Subsystem Design	**X-band only system** *vs*. dual-band (Ka and X) system	• A Ka-band downlink system requires a pointing accuracy of 0.06°, which is not possible to reliably achieve with a spin stabilization. • An X-band downlink system requires a pointing accuracy of only 0.2°, which is more readily achievable with spin-stabilization. • A dual-band system is more complex, driving a higher flight system dry mass and mission cost.
Thermal Subsystem Design	**Pump-driven fluid loop** *vs*. ‘thermos-bottle’ design	• Thermal-loop utilizes RTG waste heat to keep all components within operational temperature limits with minimal additional heater power. • A thermos-bottle design, similar to New Horizons, relies on near constant bus dissipation. The requisite, large RF amplifier drives the need for power-intensive make-up heaters when not downlinking.
Downlink CONOPS at Uranus	**Battery use for all downlink** *vs*. power-positive comms mode	• A spin-stabilized, X-band-only flight system requires a toroidal beam antenna and very large RF amplifier to close the link during UOI burn (a critical event). • Multiple heavy, expensive RTGs would be required to enable a power-positive mode with the selected RF amplifier.
RTG Selection	**One NG-RTG** *vs*. two MMRTGs	• Given the results of the above trades either RTG configuration was deemed to be sufficient to meet mission objectives. • The NG-RTG solution was chosen to enable an additional Gb of science data on each orbit (2.5 Gb/orbit with one NG-RTG vs. 1.5 Gb/orbit with two MMRTGs).
Battery Size	Many sizes considered **(11.7-kWhr (BOL) Dragonfly analogy baselined)**	• A minimum battery size of 1800 Whr (BOL) is required to complete the science portion of the orbit while maintaining a depth of discharge above 50%. • The maximum length and total number of downlink opportunities required per orbit is driven by the battery size. The minimum battery size referenced above resulted in a maximum downlink opportunity length of about 3 hr and required more than 40 contacts (and battery charge/discharge cycles) per 22-day orbit. • Assuming a battery size analogous in size to Dragonfly increased the maximum downlink opportunity length to about 12 hr, requiring only 11 ground contacts per orbit. • A mass savings opportunity could be realized with a smaller battery if a shorter maximum downlink opportunity length is accepted.

## Mission Life-Cycle Cost

The cost estimate prepared for the PERSEUS mission concept is at CML 4 (e.g., Wheatcraft and Lewis 2018). The payload and spacecraft estimates capture the resources required for a preferred point design and take into account subsystem level mass, power, and risk. The cost estimate also takes into account the technical and performance characteristics of components. Estimates for the work breakdown structure (WBS) elements whose costs are primarily determined by labor – e.g., Science (WBS 4), Mission Operations (WBS 7), and Ground Data System (WBS 9) – take into account the Phase A–D schedule and Phase E timeline.

The result is a mission estimate that is comprehensive and representative of expenditures that might be expected if the PERSEUS mission is executed as described. The PERSEUS Phase A–F mission cost, including unencumbered reserves of 50% (B–D) and 25% (E–F), is $1.63B in fiscal year 2025 dollars (FY$25), as shown in Table 7. Excluding all launch vehicle-related costs, the PERSEUS Phase A–D mission cost is $940M FY25.

**Table 7:** Estimated Phase A–F PERSEUS mission cost by Level-2 WBS element

PERSEUS Mission Cost Estimate (FY25$M)					
WBS	Description	Phase A-D	Phase E-F	Total	Notes
	Phase A	7.0	-	7.0	Assumption based on previous studies
1/2/3	Project Management (PM)/ Systems Engineering (SE)/Mission Assurance (MA)	77.0	-	77.0	A-D: Wrap factor based on recent New Frontiers and APL missions; E-F Bookkept with WBS 7
4	Science	22.8	92.8	115.7	Cost per month of recent New Frontiers and APL missions
5	Payload	118.6	-	118.6	Parametric and analogy-based estimates
6	Spacecraft	302.1	-	302.1	Estimated via parametric models
7	Mission Operations	25.9	277.6	303.5	Cost per month of recent New Frontiers and APL missions
8	Launch Vehicle	225.0	-	225.0	Falcon Heavy Expendable placeholder
9	Ground Data Systems	12.4	1.9	14.3	Rough order of magnitude (ROM) bottoms-up estimate (BUE)
10	Integration & Testing (I&T)	63.4	-	63.4	APL historical I&T percentage of hardware (including testbeds)
	**Subtotal**	**854.2**	**372.3**	**1,226.5**	
	Reserves	311.1	93.1	404.2	50% B-D, 25% E-F, excludes WBS 8
	**Total with Reserves**	**1,165.3**	**465.4**	**1,630.7**	
	**Total with Reserves, excl. Launch Vehicle**	**940.3**	**465.4**	**1,405.7**	

### Mission Ground Rules and Assumptions

Mission costs are reported using the Level-2 (and Level-3 where appropriate) WBS provided in NPR 7120.5F (NASA 2021b). All cost estimates are reported in FY$25. The NASA New Start Inflation Index (NASA 2016) was used to adjust historical cost, price data, and parametric results to FY$25 where necessary. The mission does not require Technology Development dollars to advance components to TRL 6 because all PERSEUS mission components are already at TRL 6 or higher. A Mod-1 NG-RTG is included in WBS 6 estimate and costed at $70M per the 2022 Planetary Science and Astrobiology Decadal Study Ground Rules (NASA 2021c). A launch vehicle cost estimate of $225M is held in WBS 8 and assumes a SpaceX Falcon Heavy Expendable launch vehicle.

Phase B–D cost reserves are calculated as 50% of the estimated costs of all components excluding the launch vehicle. Phase E–F cost reserves are calculated as 25% of the estimated costs of all Phase E elements excluding NASA Deep Space Network (DSN) charges.

### Cost Benchmarking

**Fig. 11:**
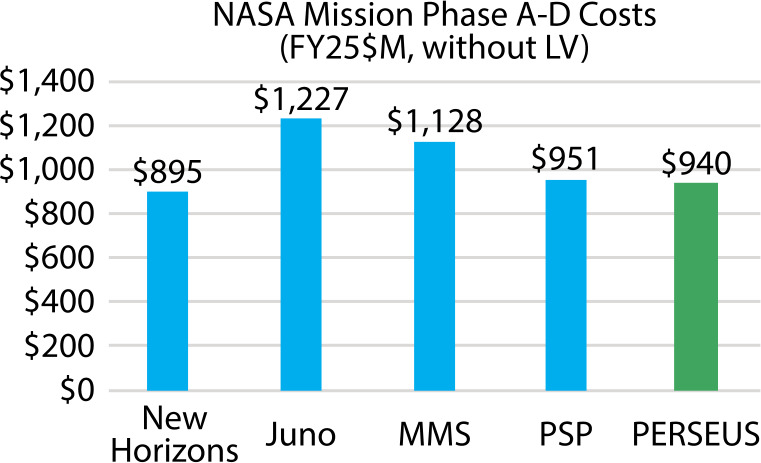
PERSEUS Phase A-D cost versus comparable NASA missions

The cost and scope of the PERSEUS concept corresponds well with the previous NASA Heliophysics and Planetary Science missions shown in Fig. 11. The estimated cost to develop PERSEUS compares favorably to these NASA missions with an average cost of $1,050M. Excluding the launch vehicle, the Phase A-D PERSEUS estimate of $940M FY25$ would bring it roughly in the same mission cost range as the most recently-launched Heliophysics Solar Terrestrial Probes (STP) and Living With a Star (LWS) missions.

### Methodology and Basis of Estimate

The PERSEUS CML 4 mission cost estimate is a combination of high-level parametric and analog techniques and incorporates a wide range of uncertainty in the estimating process. No adjustments were made to remove the historical cost of manifested risk from the heritage data underlying the baseline estimate. Therefore, before reserves are applied, the estimated costs already include a historical average of the cost of risk. This approach is appropriate for capturing risk and uncertainty commensurate with the early formulation stages of a mission. The following describes the basis of estimate for each element.

#### WBS 1, 2, 3 – Project Management (PM), Systems Engineering (SE), Mission Assurance (MA)

Because these functions depend on multiple mission- and organization-specific characteristics (Hahn 2014, 2015a, 2015b), cost analogies to comparable historical missions are preferred over cost model outputs, which do not take the mission into account. Existing analyses demonstrate that hardware costs are a reliable predictor of these critical mission function costs. APL has conducted thorough and rigorous analyses of Project Management (PM)/Systems Engineering (SE)/Mission Assurance (MA) costs, both for historical APL missions and for analogous missions. The PM/SE/MA estimate for PERSEUS relies on APL’s analysis of historical PM, SE, and MA practices on Van Allen Probes, Parker Solar Probe, and New Horizons. In particular, Van Allen Probes and Parker Solar Probe included costs associated with current NASA requirements (e.g., Earned Value Management System (EVMS), NPR 7120.5F (NASA 2021b). The total PERSEUS mission PM/SE/MA cost is 15.9% of the flight system (i.e., payload, spacecraft, I&T). This percentage is allowed to vary along with hardware costs as part of the mission cost risk analysis, discussed in Sect. 4.4, to capture uncertainty (particularly given the current CML-4-level design phase).

#### WBS 4 – Science

This element covers the managing, directing, and controlling of the science investigation. It includes the costs of the Principal Investigator, Project Scientist, science team members, and related activities. The Phase A–D and E–F science estimates are analogous estimates based on the cost per month of New Horizons, MESSENGER, Cassini, Dragonfly, OSIRIS-Rex, and Juno. New Horizons is APL’s most-recently flown New Frontiers mission (with an overall cost and long-duration cruise similar to PERSEUS) and MESSENGER is a recent historical data point for planetary orbital science. The analogy costs are representative of expenditures for science on a typical New Frontiers-scale mission. The estimate reflects the staffing levels needed to create various data products as well as to ensure closure to science objectives.

#### WBS 5 – Payload

The WBS 5 estimate includes a science payload of eight (8) instruments and payload-level PM/SE/MA (Table 8). The 8.2% cost-to-cost factor for estimating payload PM/SE/MA costs is based on the Van Allen Probes, New Horizons, MESSENGER, and Parker Solar Probe payload suite cost data with PM/SE/MA costs estimated as a percentage of the payload hardware. Technical management and systems engineering costs for individual instruments are carried in their respective instrument development costs.

**Table 8:** WBS 5 estimate in FY$25M

PERSEUS Mission Estimate (FY25$M)			
WBS	Description	Total	Notes
**5**	**Payload**	**118.6**	**Parametric and analogy-based estimates**
5.1	Payload PM/SE/MA	9.0	Based on Van Allen Probes, New Horizons, MESSENGER, Parker Solar Probe percent of hardware
5.2	JADE	35.2	Average of NICM 9/SEER Space estimates
5.3	CHEMS	21.7	
5.4	EPI-Lo	12.6	
5.5	MAG (FGM + SCM)	4.4	
5.6	WAVES	5.9	
5.7	WAVES & MAG DPU	6.7	
5.8	JunoCam	8.9	
5.9	Alice	14.2	

Given the early design phase of PERSEUS, multiple approaches were used to estimate each instrument to capture the potential range in cost. This includes two parametric estimates that rely on different sets of input variables (i.e., SEER Space (e.g., Friz et al. 2018) and NICM 9 (Mrozinski 2021)). An average of the two parametric estimates is used as the point estimate to prevent estimate bias (high or low). These estimates are subject to a cost risk analysis (discussed in Sect. 4.4) to further quantify uncertainty. No technology development is required for the payload. All payload elements in this estimate are assessed at TRL 9.

#### WBS 6 – Spacecraft

The WBS 6 estimate includes the spacecraft bus, flight software, component engineering, and RTG (Table 9). Spacecraft PM/SE/MA is carried in WBS 1, 2, and 3 consistent with APL in-house builds (Hahn 2014, 2015a, 2015b). The basis of estimate relies primarily on parametric models and APL historical cost per kilogram at the subsystem level. An average of three parametric estimates and the APL cost per kilogram is used as the point estimate to mitigate estimate bias (high or low). SEER Space is one of the primary estimating methodologies because it was designed specifically for missions in early formulation stages. TruePlanning and SEER H (e.g., Friz et al. 2018) are also utilized as they provide cost estimates at the component level. No technology development is required for the spacecraft.

**Table 9:** WBS 6 costs in FY$25M

PERSEUS Mission Estimate (FY25$M)			
WBS	Description	Total	Notes
**6**	**Spacecraft**	**302.1**	**Estimated via parametric models**
6.1	Mechanical	27.1	All subsystem estimates use the average of: SEER and PRICE TruePlanning model outputs, as well as APL historical cost per kilogram. FSW and Component Engineering estimates are based on averages from larger APL historical missions.
6.2	Propulsion	31.5	
6.3	Avionics	17.1	
6.4	Power	44.1	
6.5	Guidance and Control	15.1	
6.6	Thermal	16.0	
6.7	Communications	40.3	
6.8	Harness	6.2	
6.9	Flight Software	17.0	
6.A	Component Engineering	17.7	
6.X	NG-RTG Mod-1	70.0	Per 2022 Planetary Science and Astrobiology Decadal Study Ground Rules

#### WBS 7 – Mission Operations and WBS 9 – Ground Data Systems (GDS)

The PERSEUS mission operations estimate includes mission operations planning and development, network security, data processing, and mission management. The pre- and post-launch mission operations estimates are based on the cost per month of past and ongoing deep-space missions (e.g., New Horizons, Dragonfly, Juno, and OSIRIS-Rex) with comparable scope and complexity. The GDS estimate is a rough order of magnitude bottoms-up estimate from the APL Space Mission Operations group. The PERSEUS GDS provides full life cycle support for subsystem test, observatory I&T, hardware simulator control, and flight operations. The cost estimate is based on extensive reuse of ground software from Parker Solar Probe, IMAP, and DART via APL’s Mission Independent Ground Software (MIGS), as well as use of the existing APL Mission Operations Center (MOC).

WBS 7 also includes set up costs for DSN usage based on prior missions. However, cost estimates for access to the DSN infrastructure needed to transmit and receive mission and scientific data are *not* included in the PERSEUS mission budget and the cost tables provided here. These charges are estimated at $21M using the Jet Propulsion Laboratory (JPL) DSN Aperture Fee tool (JPL 2023) and cover pre- and post-contact activity for each linkage.

#### WBS 8 – Launch Vehicle and Services

The mission requires a launch vehicle that does not correspond with any of the options currently described in the 2022 Planetary Science and Astrobiology Decadal Survey Ground Rules (NASA 2021c). As such, the figures used in this estimate are based on an evaluation of current best estimates of the cost of the capability that would be required. The price of a launch vehicle with Falcon Heavy Expendable-type capabilities, based on past pricing for NASA missions of Evolved Expendable Launch Vehicles (EELVs), would be approximately $225M for a launch using a standard sized fairing.

#### WBS 10 – System Integration and Testing (I&T)

This WBS element covers the efforts to assemble and test the spacecraft and instruments. The PERSEUS I&T effort is estimated as 12.7% of the hardware. This percentage is based on a detailed analysis of cost actuals from previous APL missions, including MESSENGER, New Horizons, STEREO, Van Allen Probes, and Parker Solar Probe. This percentage is allowed to vary along with hardware costs as part of the mission cost risk analysis to capture the risk historically manifested during I&T.

### Confidence, Cost Reserves, and Cost Risk

The cost risk ranges by major WBS element as inputs for the PERSEUS probabilistic cost risk analysis to quantify total cost risk are described below. The launch vehicle costs are excluded from the risk analysis.

**Fig. 12:**
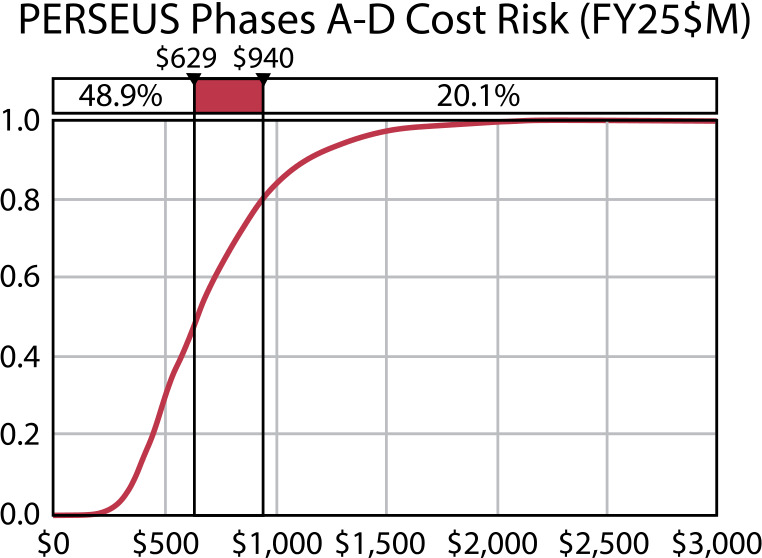
S-curve summary. The point design presented here is estimated at $629M with ∼49% confidence. The total mission cost (not including the launch vehicle) with reserves is estimated at $940M with 80% confidence

The PERSEUS cost estimate includes unencumbered cost reserves of 50% of the estimated costs for all Phase B–D elements except for the launch vehicle. A probabilistic cost risk analysis shows 80% confidence that the Phase A–D mission is achievable within the estimated costs of this study (Fig. 12). The high confidence level is driven primarily by the large cost reserves for this CML-4 concept. Given a typical competitive pre-Phase A NASA environment with 25% reserves on Phase A–D elements, the probabilistic cost risk analysis shows 67% confidence that the Phase A–D mission cost would be achievable. A 50th- to 70th-percentile confidence level is expected and reasonable for a pre-Phase A concept with this level of reserves.

A coefficient of variation (standard deviation/mean) of approximately 46% indicates appropriate levels of conservatism given the early formulation phase. The model confirms the point estimate and provides a reasonable basis for the PERSEUS CML-4 study.

## Summary and Conclusions

The PERSEUS mission concept was formulated to investigate the feasibility and potential scope of a dedicated, standalone Heliophysics orbiter mission to study the complex and dynamic magnetosphere of Uranus and the interactions between magnetospheric plasma, particles, and energy with the system’s atmosphere, rings, and moons. From the planet’s tilted and offset, rapidly-rotating non-dipolar magnetic field to its seasonally-extreme interactions with the solar wind to its unexpectedly intense electron radiation belts, Uranus hosts a range of outstanding and compelling mysteries relevant to NASA’s Heliophysics Division. While the exploration of planets other than Earth has largely fallen within the purview of NASA’s Planetary Science Division, many targets, like Uranus, hold immense scientific value and interest to the space physics community. Exploring and understanding Uranus’s magnetosphere is critical to make fundamental gains in magnetospheric physics and understanding of potential exoplanetary systems and to test the validity of our understanding of various processes – such as magnetospheric dynamics, moon-magnetosphere interactions, magnetosphere-ionosphere coupling, and solar wind-planetary coupling – and whether they are universal across planetary magnetospheres or a result of unique characteristics of certain systems. The PERSEUS mission concept study presented here at CML 4, comprises a realistic particles and fields payload with a UV spectrometer and EPO camera that provides closure to a range of space physics science objectives in a reliable and mature spacecraft and mission design architecture. The interplanetary trajectory targets at 2031 baseline launch and utilizes a JGA to reach Uranus in under 13 years. The mission CONOPS borrows a passive cooling loop architecture from the Dragonfly mission that relies heavily on hibernation but enables the mission to close with the use of only a single Mod-1 NG-RTG. The overall mission cost for the baseline two-year prime mission is estimated to cost $1.4B with reserves (not including the LV costs), which is comparable to the costs of recent Heliophysics missions such as Parker Solar Probe.
